# Mapping and QTL Analysis of Gynoecy and Earliness in Bitter Gourd (*Momordica charantia* L.) Using Genotyping-by-Sequencing (GBS) Technology

**DOI:** 10.3389/fpls.2018.01555

**Published:** 2018-10-31

**Authors:** P. Gangadhara Rao, Tusar Kanti Behera, Ambika B. Gaikwad, Anilabh Das Munshi, Gograj Singh Jat, G. Boopalakrishnan

**Affiliations:** ^1^Division of Vegetable Science, ICAR-Indian Agricultural Research Institute, New Delhi, India; ^2^ICAR-National Bureau of Plant Genetic Resources, New Delhi, India

**Keywords:** bitter gourd, earliness, genotyping-by-sequencing, gynoecy, mapping, sex ratio

## Abstract

A high-density, high-resolution genetic map was constructed for bitter gourd (*Momordica charantia* L.). A total of 2013 high quality SNP markers binned to 20 linkage groups (LG) spanning a cumulative distance of 2329.2 cM were developed. Each LG ranging from 185.2 cM (LG-12) to 46.2 cM (LG-17) and average LG span of 116.46 cM. The number of SNP markers mapped in each LG varied from 23 markers in LG-20 to 146 markers in LG-1 with an average of 100.65 SNPs per LG. The average distance between markers was 1.16 cM across 20 LGs and average distance between the markers ranged from 0.70 (LG-4) to 2.92 (LG-20). A total of 22 QTLs for four traits (gynoecy, sex ratio, node and days at first female flower appearance) were identified and mapped on 20 LGs. The gynoecious (*gy-1*) locus is flanked by markers TP_54865 and TP_54890 on LG 12 at a distance of 3.04 cM to TP_54890 and the major QTLs identified for the earliness traits will be extremely useful in marker development and MAS for rapid development of various gynoecious lines with different genetic background of best combiner for development of early and high yielding hybrids in bitter gourd.

## Introduction

Bitter gourd (*Momordica charantia* L.; 2*n* = 22) is an economically important vegetable crop belonging to the subtribe Thalidianthinae, tribe Joliffieae, subfamily Cucurbitoideae and family Cucurbitaceae (Jeffrey, 1980; De Wilde and Duyfjes, 2002). It is widely cultivated in India, China, Malaysia, Africa, and South America (Singh, 1990; Raj et al., 1993). Indian bitter gourd has wide phenotypic variation with respect to growth habit, maturity, fruit shape, size, color, and surface texture (Robinson and Decker-Walters, 1997) and sex expression (Behera et al., 2006). Fruits with seeds of bitter gourd are consumed at immature stage and possess medicinal properties such as anti-diabetes (e.g., India, China, and Central America; Chen et al., 2003), hypoglycaemic compounds (Jayasooriya et al., 2000), anti-carcinogenic and hypercholesterolemic (Ganguly et al., 2000; Ahmed et al., 2001), anti-HIV activity (Lee et al., 1995) and also contain charantin (Yeh et al., 2003), momorcharin (Leung et al., 1997), momordicosides A and B (Okabe et al., 1980). Bitter gourd possesses comparatively high concentrations of ascorbic acid and iron than other cucurbitaceous vegetable crops (Behera, 2004).

Like other cucurbitaceous vegetable crops, hybrids in bitter gourd offer opportunity of earliness, high yield, and quality improvement besides better capacity to counteract biotic and abiotic stresses. The hybrid development in bitter gourd may be limited because of traditional practice of hand pollination, which requires lot of labor and time, but development of hybrids using stable gynoecious lines or predominately gynoecious lines would be highly useful. The predominant sex form in bitter gourd is monoecious, however, gynoecious sex form has been reported from India, Japan, and China (Ram et al., 2002; Behera et al., 2006; Iwamoto and Ishida, 2006). In bitter gourd, gynoecism is under the control of a single recessive gene (*gy-1*) (Ram et al., 2006; Behera et al., 2009; Matsumura et al., 2014), whereas two pairs of genes reported by Cui et al. (2018). The flowering traits like days to first pistillate flower appearance, node at first pistillate flower appearance and staminate: pistillate (♂:♀) flower ratio (sex ratio) are directly related to earliness and fruit yield. Production of hybrid seeds in bitter gourd is highly expensive because it is done mainly through hand pollination. But utilization of a gynoecious line would be more economical and easier method (Behera et al., 2009). Since gynoecious parent produces only female flowers, the open pollinated seeds produced in these plants will be F_1_ hybrid. It reduces the cost of male flower pinching and hand pollination (Behera et al., 2009).

Conventional phenotypic selection for high and stable yield requires the evaluation of yield in multiple environments over several seasons; which is very expensive and time consuming (Yuan et al., 2002). In contrast, marker assisted selection (MAS) certainly accelerates the breeding process and powerful tool for selecting traits such as gynoecism. The scarcity of polymorphic molecular markers in the public database has hindered genetic mapping and the application of molecular breeding in bitter gourd. The molecular basis of agronomically important traits remains unexplored to date and decisive linkage map has not been reported in bitter gourd. Various multi-locus dominant DNA markers such as RAPD (Dey et al., 2006; Paul et al., 2010), ISSR (Singh et al., 2007), and AFLP (Gaikwad et al., 2008) have been reported for genetic analyses of bitter gourd. SSRs are known to have high heterozygosity values and are more informative than dominant DNA markers (Powell et al., 1996). However, the number of microsatellite markers available in bitter gourd is few. Among the 70 SSR markers reported, 16 have been developed using FIASCO technique (Guo et al., 2012; Ji et al., 2012), 11 through genomic library enrichment (Xu et al., 2011) and 43 through cross-species transferability from other cucurbits (Chiba et al., 2003; Watcharawongpaiboon and Chunwongse, 2008; Xu et al., 2011). It is established that greater number of markers are necessary for the development of genetic map and MAS (Tang et al., 2007). A novel set of 160 microsatellite markers has been developed in *Momordica* species through sequencing of small insert genomic library enriched for 10 different repeat motifs (Saxena et al., 2015), but they showed polymorphism across the *Momordica* species and less variation within the *M. charantia* genotypes.

The first genetic map and positions of major fruit trait loci of bitter melon were worked out by Kole et al. (2012). An extensive genetic linkage map was constructed for bitter gourd *via* the study of F_2:3_ progenies derived from two cultivated inbred lines (Wang and Xiang, 2013). Matsumura et al. (2014) identified SNP marker, GTFL-1 that was linked to the gynoecious locus at a distance of 5.46 cM by using RAD-seq (restriction-associated DNA tag sequencing) analysis. Bitter gourd (*M. charantia*) draft genome sequence (Urasaki et al., 2017) of a monoecious inbred line, OHB3-1, was analyzed through Illumina sequencing and *de novo* assembly, scaffolds of 285.5 Mb in length were generated corresponding to ∼84% of the estimated genome size of bitter gourd (339 Mb). Draft genome sequence of bitter gourd revealed that, the MOMC3_649 in bitter gourd was presumed to be an ortholog of *CmAcs11* (female flower determination in melon) and two proteins (MOMC46_189, MOMC518_1) were found in bitter gourd similar to CmAcs-7 (unisexual flower development in melon) grouped in the same clade in the phylogenetic tree sequence (Urasaki et al., 2017). Cui et al. (2018) did the RAD-based genetic map for anchoring scaffold sequences and identified QTLs for gynoecy, first flower node, female flower number, fruit epidermal structure and fruit color in bitter gourd.

Elshire et al. (2011) have developed simple and highly multiplexed genotyping by sequencing (GBS) approach for population studies, germplasm characterization and mapping of desired traits in diverse organisms. GBS depends on high-throughput, next-generation sequencing (NGS) of genomic subsets targeted by restriction enzymes (REs) at low cost per sample and an advantage in crops like bitter gourd that lacks a complete genome sequence, a reference map need to be developed only around the restriction sites (Elshire et al., 2011). The consensus of read clusters across sequence tagged sites becomes the reference in case of crops that lack reference genome sequence. The innovative GBS approach offers an ultimate MAS tool to accelerate crop improvement program (He et al., 2014). However, to date there is very scattered information related to QTL mapping for horticultural traits in bitter gourd. Keeping the aforesaid information in view, the present experiment was undertaken with objective to map gynoecious (*gy-1*) gene, sex ratio, and earliness related traits in bitter gourd for further utilization in bitter gourd crop improvement.

## Materials and Methods

### Plant Material for Genotyping by Sequencing (GBS)

The genetic material used for mapping of gynoecious and earliness involved 90 F_2_ segregated population and 65 F_2:3_ families (individual plants of F_2_ selfed) derived from a cross between DBGy-201 (PVGy-201) and Pusa Do Mousami (PDM). It is very difficult in self-fertilizing and seed production of gynoecious lines to F_2:3_ population. The female parent PVGy-201 is a gynoecious line (100% female flowers) and first pistillate flower appear at 7th node on 33 days after planting; whereas male parent Pusa Do Mousami is a monoecious plant (♂:♀ is 17:1) and first pistillate flower appear at 13th node on 60 days after planting. The F_2_ plants along with the two parents were planted at vegetable research farm of Indian Agricultural Research Institute (IARI), New Delhi, India during spring summer (February–May) 2015 for phenotyping the qualitative traits like gynoeciousim and sex ratio. A total of 65 F_2:3_ families along with two parents were planted during spring summer (February–May) 2016 to study the quantitative traits like node and days to first pistillate flower appearance. About 20 F_3_ seeds from each F_2_ plant were sown in single row with three replications and recommended agronomic practices were undertaken for the healthy crop.

### Phenotyping

Phenotyping of parental lines, F_1_, back crosses, F_2_ and F_2:3_ populations was performed to study the inheritance and mapping of traits related to sex ratio (Supplementary Table S1) and earliness. The sex (♂ or ♀) of 20 flowers each in parents, F_1_, F_2_, F_2:3_ and backcross populations was investigated. Plants carrying only the female flowers were defined as gynoecious plants, while the other plants were classified as monoecious plants in this study. The data was recorded on individual basis, 20 plants in each parent, 30 plants each in F_1_s, BC_1_P_1_ and BC_1_P_2_, 90 plants in F_2_ and 65 plants in F_2:3_ populations.

### Genotyping

#### Genomic DNA Extraction and Quantification

Genomic DNA was extracted from leaf tissue by following modified CTAB method (Saghai-Maroof et al., 1984). The quantity and quality of extracted genomic DNA was checked by spectrophotometer (NanoDrop 8000; Thermo Fisher Scientific). An estimated quantity of 100 ng/μL of total genomic DNA was used to prepare each library.

### GBS Library Preparation and Sequencing

#### Restriction Enzyme (RE)

Two major points considered while choosing REs; firstly, REs that cuts to leave overhangs of 2 to 3 bp, secondly, REs that do not cut frequently in the major repetitive fraction of the genome. Different REs like *Ape*KI, *Eco*T22I, *Msp*I, and *Pst*I are screened to choose most appropriate RE for bitter gourd GBS library preparation. Among these *Ape*kI enzyme was given best library fragment distribution and hence, *Ape*kI was chosen for library preparation for all bitter gourd samples (Supplementary Data Sheet S1). 96 plex library preparation protocol was designed according to Elshire et al. (2011; Supplementary Data Sheet S2).

#### Adapters for GBS

Two different types of adapters were used in this protocol (Elshire et al., 2011). The “barcode” adapter terminates with a 5 to 10 bp barcode on the 3′ end of its top stand and a 3 bp overhang on the 5′ end of its bottom strand that is complementary to the “sticky” end generated by *Ape*KI (CWG). The sequences of the two oligonucleotides comprising the barcode adapter are:

5′-ACACTCTTTCCCTACACGACGCTCTTCCGATCTxxxx and
5′-CWGyyyyAGATCGGAAGAGCGTCGTGTAGGGAAAGAGTGT

where “xxxx” and “yyyy” denote the barcode and barcode complement and sequences.

The second, or “common,” adapter has only an *Ape*KI-compatible sticky end:

5′-CWGAGATCGGAAGAGCGGTTCAGCAGGAATGCCGAG and
5′-CTCGGCATTCCTGCTGAACCGCTCTTCCGATCT

For preparing each library we have used 94 different barcodes for tagging samples, which have variable length from 5 to 10 nucleotides.

#### Illumina Sequencing

The final 93 libraries (90 F_2_ and each one from two parents and F_1_) were sequenced using Illumina True Seq Version 3 single end sequencing chemistry with read lengths of 150 bp on HiSEQ 2000 Platform. Ninety four samples (plus a blank negative control) were sequenced per lane. The library was prepared for GBS by following the Elshire et al. (2011) protocol and the complete genomic data was deposited at NCBI (the SRA number was SUB4509570 and the Bioproject ID was PRJNA493717).

#### Raw Sequence Data Processing

The reads were filtered following Elshire et al. (2011) protocol; perfectly matched one of the barcodes and the expected four-base remnant of the *Ape*KI cut site (CWGC), no adapter dimers and reads with no “NS” (minimum Qscore of 10) across the first 72 bases. The final size of raw data from all libraries was 18.4 GB. As bitter gourd was not having reference genome, sequence reads from raw data FASTQ file were processed through *de novo* GBS analysis pipeline as implemented in UNEAK (Supplementary Data Sheet S3). Software for sequence filtering and the mapping analysis is a part of the TASSEL package and is available on Source Forge^1^ (Supplementary Data Sheet S4).

#### Construction of Genetic Linkage Map

The genotypic data matrix was generated based on scoring pattern observed with all polymorphic SNP markers. The generated matrix data was integrated with all polymorphic SNP markers used as an input file in JOINMAP^®^ 4.1 program (Van Ooijen, 2011) for construction of linkage map. The χ^2^ test was performed for identification of markers with aberrant segregation (*p* < 0.05) by calculating the locus genotyping frequencies in JOINMAP^®^4.1. Linkage groups (LGs) were constructed by grouping of markers at a minimum independence LOD threshold of 3.0 and a maximum of 10.0 with a step up of 0.5. The groups were converted to maps at LOD using regression algorithm with the following settings: linkages with recombination frequency (<0.49), LOD (>0.01) threshold for removal of loci with respect to jumps in goodness-of-fit (5.0) and performing a ripple after adding 2 loci. Distance was calculated using Kosambi’s mapping function and LGs were drawn with help of Map Chart.

#### QTL Analysis

Quantitative trait loci analysis was carried out on the set of 90 F_2_ individuals with phenotypic data for sex ratio and earliness (both F_2_ and F_3_ populations). The genotypic data consisted of marker loci. QTLs were detected with the WinQTL Cartographer v2.5 (Wang et al., 2012) software by composite interval mapping (CIM) (Zeng, 1993, 1994). The statistical significance thresholds used to declare the presence of QTLs were determined by 1,000 random permutations with a genome-wide type I error rate of 5% (*p* = 0.05) (Doerge and Churchill, 1996). The 95% confidence intervals of the QTL locations were determined by one LOD intervals surrounding the QTL peak (Mangin et al., 1994). Additive effect of the detected QTLs was also estimated by the WinQTL Cartographer v2.5. The *R*^2^ value from this analysis was accepted as the percent phenotypic variance explained by the locus.

## Results

### Sequencing and Identification of SNPs

Raw data of Illumina sequence of all 93 libraries was 18.4 GB. The total 93 pooled, barcoded samples (each sample from 90 F_2_ individuals, F_1_ and two parents) have generated 93,926 SNP sites. After excluding SNPs that were monomorphic in the F_2_ population, those non-biallelic, with more than 40% of missing data, with minimum allele frequency (MAF) 20%, 4003 SNPs remained. The SNPs were again filtered to remove heterozygote SNPs in both parents and finally high quality 2013 SNPs were identified and used for linkage map construction.

### Linkage Map Construction

**FIGURE 1:**
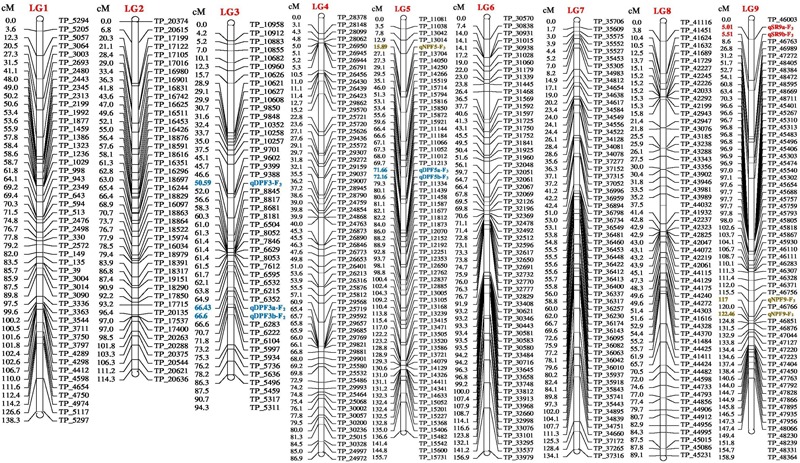
Linkage map of bitter gourd of a cross (DBGy-201 × Pusa Do Mousami) using F_2:3_ population along with peak positions of quantitative trait loci (QTLs) (LG 1-9).

**FIGURE 2:**
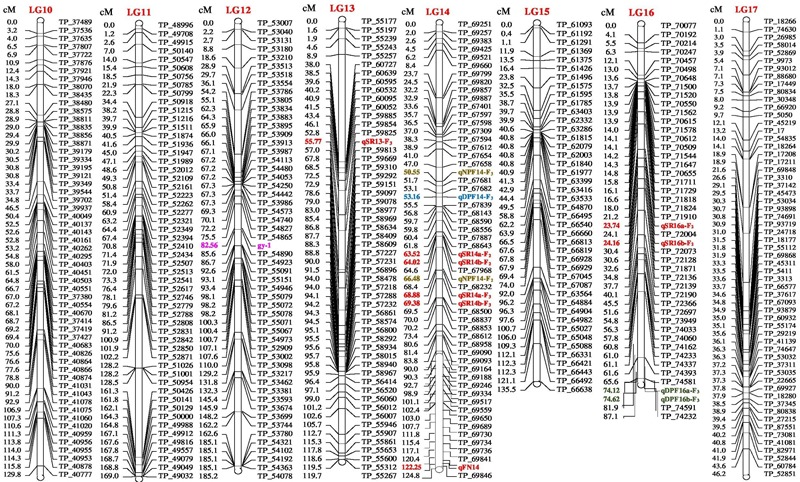
Linkage map of bitter gourd of a cross (DBGy-201 × Pusa Do Mousami) using F_2:3_ population along with peak positions of QTLs (LG 10-17).

**FIGURE 3:**
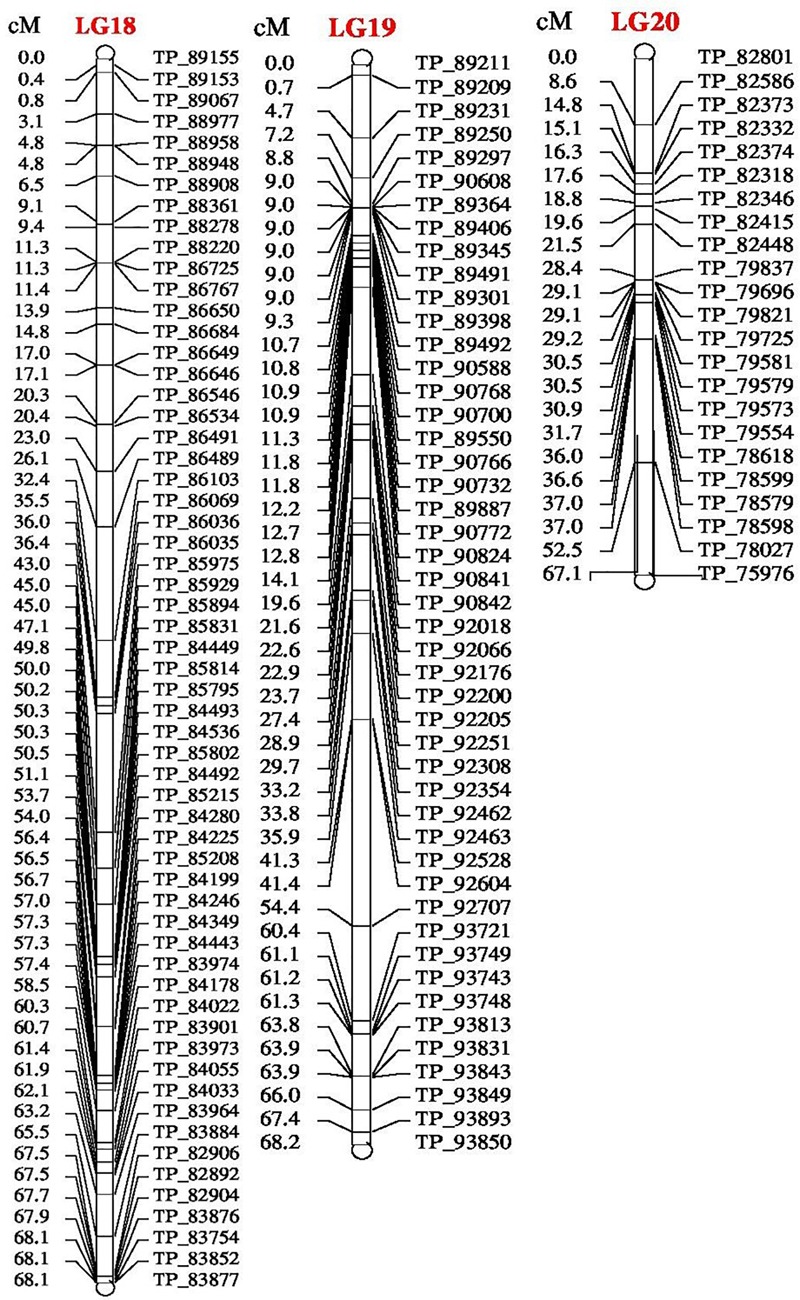
Linkage map of bitter gourd of a cross (DBGy-201 × Pusa Do Mousami) using F_2:3_ population along with peak positions of QTLs (LG 18–20).

The genetic map of the F_2_ population consisted of 2013 high quality SNP markers that binned to 20 LG (Figures 1–3). The linkage map spanned a cumulative distance of 2329.2 cM, with each LG ranging from 185.2 cM (LG 12) to 46.2 cM (LG-17) and average LG span was 116.46 cM (Table 1). The number of SNP markers mapped in each LG varied from 23 markers in LG-20 to 146 markers in LG-1, with an average of 100.65 SNPs per LG. The average distance between markers was 1.16 cM across 20 LGs and average distance between the markers ranged from 0.70 (LG-4) to 2.92 (LG-20).

**Table 1:** Summary of high density SNP marker distribution on linkage groups in bitter gourd cross DBGy-201 × Pusa Do Mousami.

Linkage group (LG)	Length (cM)	Number of SNP markers	Average distance between markers (cM)
LG 1	138.3	146	0.95
LG 2	114.3	131	0.87
LG 3	94.3	131	0.72
LG 4	86.9	125	0.70
LG 5	155.7	123	1.27
LG 6	156.9	121	1.30
LG 7	134.1	119	1.13
LG 8	89.1	116	0.77
LG 9	158.9	116	1.37
LG 10	129.8	114	1.14
LG 11	169.0	108	1.57
LG 12	185.2	106	1.75
LG 13	119.7	105	1.14
LG 14	124.8	95	1.31
LG 15	135.5	85	1.59
LG 16	87.1	84	1.04
LG 17	46.2	59	0.78
LG 18	68.1	59	1.15
LG 19	68.2	47	1.45
LG 20	67.1	23	2.92
Total	2329.2	2013	–
Average	116.46	100.65	1.16

### Phenotypic Variation in Parents, F_2_ and F_2:3_ Populations

The phenotyping of gynoecious trait was performed in F_2_ population and quantitative traits like sex ratio and earliness were performed in both F_2_ and F_2:3_ populations. Descriptive statistics (Table 2) (range, mean, variance, standard deviation, skewness, and kurtosis), co-relation for three quantitative traits are shown in Table 3.

**Table 2:** Descriptive statistics for cross DBGy-201 × Pusa Do Mousami.

S. no.	Particulars	Parents		F_1_	F_2:3_ population							
	Trait	Female	Male		Range	Mean	*SD*	Variance	Skewness	Kurtosis	CV (%)	Transgressive segregants (%)
1	Node at first pistillate flower appearance	7.25	12.75	6.13	2.50–15.75	5.30	2.33	5.44	2.03	8.38	43.96	86.15
2	Days to first pistillate flower appearance	33.65	59.50	37.85	32.63–46.63	39.13	2.61	6.80	0.39	3.54	6.67	1.54
3	Sex ratio (male: female)	0.00	17.10	2.80	0.56–21.37	6.99	6.12	37.48	1.43	3.80	87.55	13.85

**Table 3:** Pearson’s correlation coefficient of major horticulture traits of bitter gourd cross DBGy-201 × Pusa Do Mousami.

Trait	Node at first pistillate flower appearance	Days to first pistillate flower appearance	Sex ratio (male: female)
Node at first pistillate flower appearance	1.00	0.627^∗∗^	0.526^∗∗^
Days to first pistillate flower appearance		1.00	0.316^∗∗^
Sex ratio (male: female)			1.00

*^∗∗^Significant at 1% level.*

### Inheritance of Gynoecy

Inheritance pattern of gynoecy was investigated in bitter gourd in cross DBGy-201 (100% pistillate flower frequency) × Pusa Do Mousami (6% pistillate flower frequency), the F_1_ (33% pistillate flower frequency) generation all plants were monoecious which indicated it as dominant trait (Table 4). Out of total 90 plants of F_2_ population (5–100% segregation for pistillate flower frequency), 72 plants were monoecious and 18 plants gynoecious. The observed frequency of F_2_ plants fitted well in the expected ratio of 3 monoecious: 1 gynoecious as evident from the non-significant χ^2^ values of 1.20 (*P* = 0.27). The BC_1_P_1_ population segregated into 16 monoecious and 14 gynoecious plants. The observed frequency of BC_1_P_1_ plants fitted well in the expected ratio of 1 monoecious: 1 gynoecious plants with non-significant χ^2^ values of 0.13 (*P* = 0.72).

**Table 4:** Chi square (χ^2^) analysis of F_2_ population for studying inheritance pattern of gynoecious (*gy-1*) trait in bitter gourd cross DBGy-201 (Gy-1) × Pusa Do Mousami (PDM).

Parent/Cross	Monoecious	Gynoecious	Expected ratio	χ^2^-value	*P*-value
Gy-1	0	20			
PDM	20	0			
Gy-1 × PDM (F_1_)	30	0			
(Gy-1 × PDM) × Gy-1 (BC_1_P_1_)	16	14	1:1	0.13	0.72
(Gy-1 × PDM) × PDM (BC_1_P_2_)	30	0			
Gy-1 × PDM (F_2_)	72	18	3:1	1.20	0.27

### Inheritance of Sex Ratio

**FIGURE 4:**
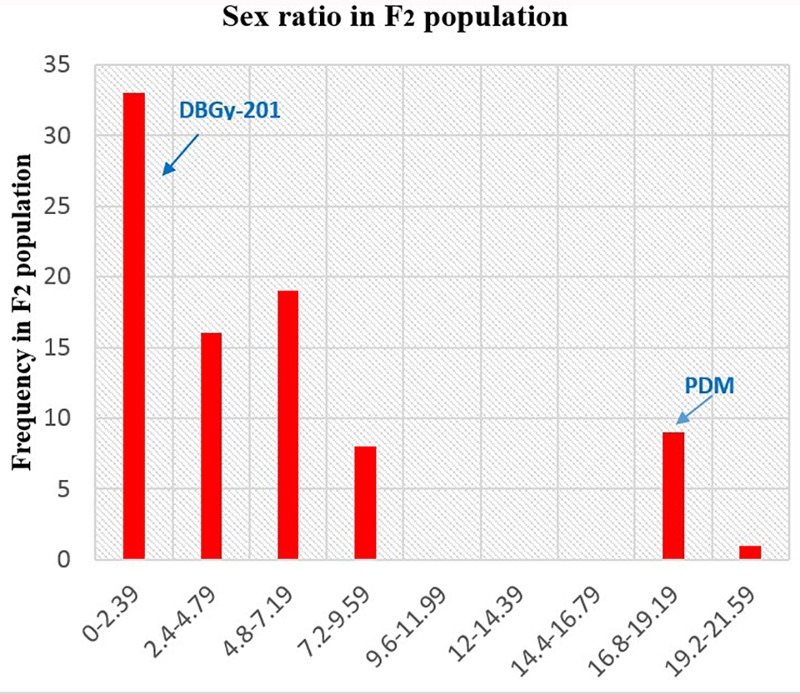
Frequency distribution pattern sex ratio in F_2_ population (DBGy-201 × Pusa Do Mousami).

**FIGURE 5:**
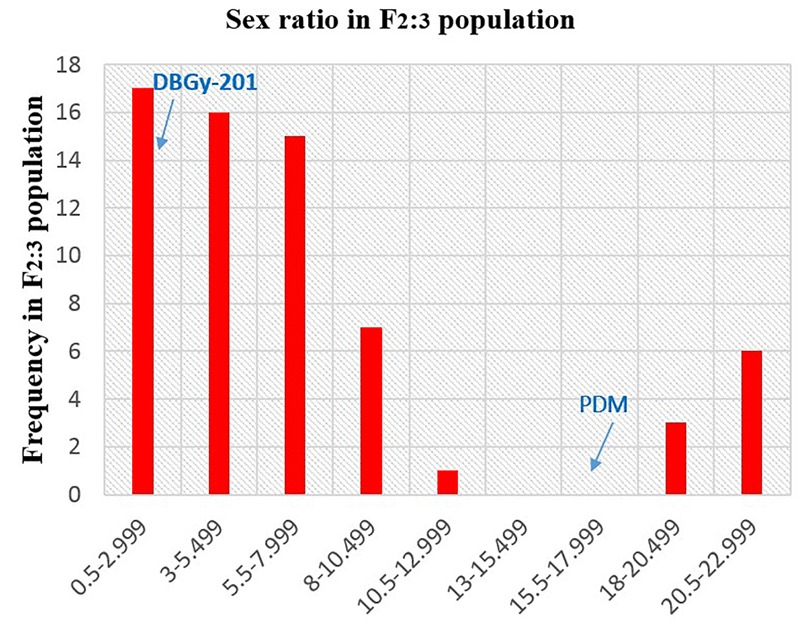
Frequency distribution pattern sex ratio in F_2:3_ population (DBGy-201 × Pusa Do Mousami).

In addition to gynoecious nature, the sex ratio (staminate:pistillate) is also an extremely important trait in cucurbits for getting higher fruit yields. The sex ratio most likely genetically controlled, but it is often affected by environmental and nutritional factors. The sex ratio of parents DBGy-201 (0 i.e., all pistillate flowers), Pusa Do Mousami (17), F_1_ (3) and segregated populations F_2_ and F_2:3_ ranged from 0 to 20 and 0.5 to 21, respectively. The transgressive segregants for sex ratio in F_2:3_ population were 13.85%. The bimodal distribution of F_2_ and F_2:3_ populations (Figures 4, 5) revealed that, the involvement of some major genes may be modified by other genes of minor effect and trait controlled by semi-quantitative genes, but the information in the histogram is too limited to conclude on this aspect.

### Inheritance of Node and Days to First Pistillate Flower Appearance

For getting higher price in the market, early picking is most important. Node and days to first pistillate flower appearance are directly contributing toward early harvesting of horticultural matured fruits. The first pistillate flower appeared for parent DBGy-201 on 7th node after 33 days of sowing and parent Pusa Do Mousami at 15th node, after 60 days of sowing. The F_1_ had first pistillate flower appeared at 6th node after 35 days of sowing. The range of segregation for node at first pistillate flower appear was 3–16 and 2.5–15.75 in F_2_ and F_2:3_ populations, respectively. For trait, days to first pistillate appearance ranged from 35 to 47 and 32.56 to 46.63 in F_2_ and F_2:3_ populations, respectively.

**FIGURE 6:**
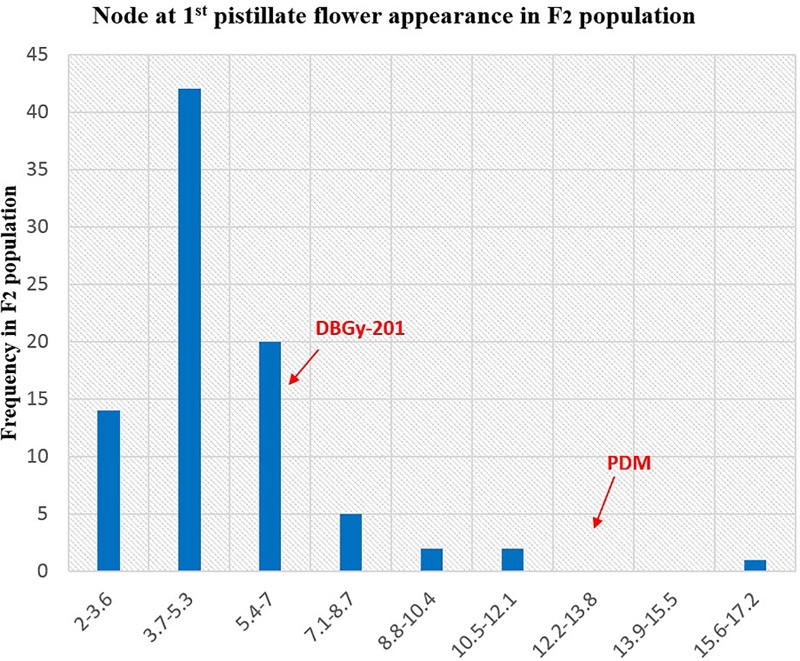
Frequency distribution pattern of node at first pistillate flower appearance in F_2_ population (DBGy-201 × Pusa Do Mousami).

**FIGURE 7:**
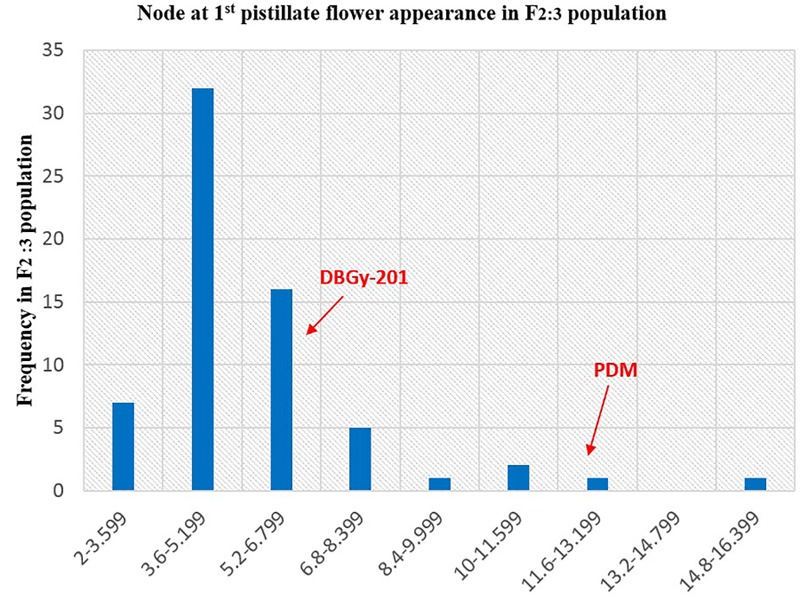
Frequency distribution pattern of node at first pistillate flower appearance in F_2:3_ population (DBGy-201 × Pusa Do Mousami).

**FIGURE 8:**
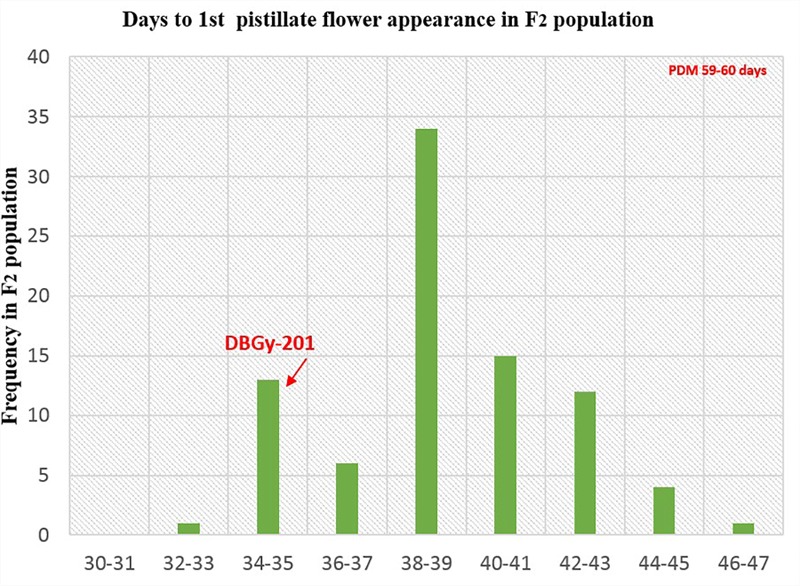
Frequency distribution pattern of days to first pistillate flower appearance in F_2_ population (DBGy-201 × Pusa Do Mousami).

**FIGURE 9:**
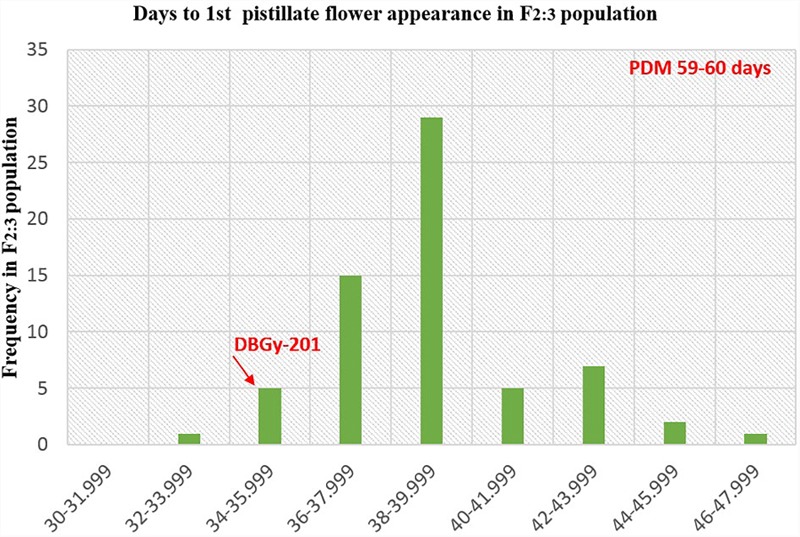
Frequency distribution pattern of days to first pistillate flower appearance in F_2:3_ population (DBGy-201 × Pusa Do Mousami).

The segregation pattern (Figures 6, 7) of trait, node at first pistillate flower appearance among F_2_ individuals and F_2:3_ families indicated that, this trait was governed by quantitative genes. For trait, node at first pistillate flower appearance, out of 90 plants in F_2_, 73 plants and in F_2:3_ out of 65 families, 55 families had shown lower than female parent PVGy-201 (<7th node; desirable), which indicated predominance of major genes toward lower node for first pistillate appearance. The segregation pattern (Figures 8, 9) of trait, days to first pistillate appearance among F_2_ individuals and F_2:3_ families indicated that this trait was governed by quantitative genes. For the trait, days to first pistillate flower appearance more plants of F_2_ individuals (55 plants out of 90 plants had in the range of 35–40 days) and F_2:3_ families (45 families out 65 families had in the range of 35–40 days) were located between the two parents, but very close to female parent (PVGy-201), this indicated predominance of major genes toward earliness for pistillate flower appearance. The transgressive segregants for node at first pistillate flower appearance in F_2:3_ population were 86.15% and for days to first pistillate flower appearance were only 1.54%.

### Mapping of Genes for Sex and Earliness Related Traits

Four most important economic traits were studied in both F_2_ and F_2:3_ mapping populations with 2013 SNP markers over 20 LGs to construct high quality genetic map. High-resolution mapping of QTLs may be used to develop reliable markers for MAS (at least <5 cM but ideally <1 cM away from the gene) and LOD score of above 2.0 or 3.0 (most commonly 3.0) was usually chosen as the significance threshold for detecting QTL (Collard et al., 2005). A QTL generally considered as major QTL if *R*^2^-value >10% and minor QTLs will usually account for <10% *R*^2^-value (Collard et al., 2005). The additive effect of a QTL with positive and negative effects indicate that, the allele which increases the trait values is in the female and male parent, respectively (Supplementary Presentation S1).

### Tagging of *gy-1* Gene

**FIGURE 10:**
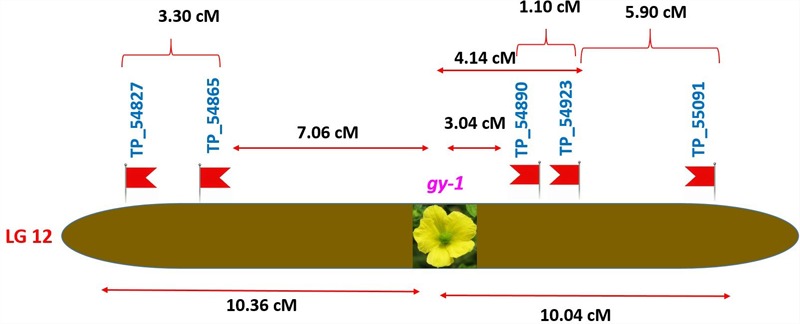
Genetic map of putative gynoecious locus (*gy-1*) on linkage group-12.

The gynoecious (*gy-1*) (Figure 10) gene was mapped on LG-12 and flanked by TP_54865 and TP_54890 markers. Marker TP_54890 was very close to *gy-1* gene at a distance of 3.04 cM (LOD = 2). The marker TP_54923 on right side of *gy-1* gene at a distance of 4.14 cM and marker TP_55091 was on right side at distance of 10.04 cM. The markers TP_54865 and TP_54827 were on left side at distance of 7.06 and 10.36 cM, respectively.

### QTL Detection for Mapping Quantitative Traits

A total of 22 QTLs related to sex ratio, node and days to first pistillate flower appearance were mapped across 20-LGs with F_2_ and F_2:3_ populations. Detailed information about all these QTLs (explained variance, LOD peaks, flanking markers, additive effects) is shown in Table 5. A total of 22 QTLs (15 QTLs with LOD > 3) were identified using CIM based on the phenotyping of both F_2_ and F_2:3_ populations. The phenotypic variation (*R*^2^%) explained by these QTLs ranged from 0.05 to 58.75% and total 11 major QTLs (*R*^2^ = >10%) were identified. Most of the QTLs identified in this study were located in the same or adjacent regions on LG-14 and LG-16, this may be due to high correlation among the traits under study (Table 3). The identified QTLs were discussed below by trait wise.

**Table 5:** QTL analysis of earliness and sex ratio in F_2_ and F_2:3_ family lines of bitter gourd.

S. no.	Trait	Generation	QTL^∗^	LG	Trait position (cM)	Flanking markers		Marker closest to peak	Closeness of marker to trait (cM)	*R*^2^ (%)	LOD	Additive effect^#^
						Left marker	Right marker					
1	Node at first pistillate flower appearance	F_2:3_	*qNPF5-F_3_*	5	15.89	TP_13014	TP_13704	TP_13014	2.99	9.51	2.9	-1.01
			*qNPF9-F_3_*	9	117	TP_46756	TP_46766	TP_46756	1.50	13.94	4	1.24
			*qNPF14-F_3_*	14	50.55	TP_67658	TP_67681	TP_67681	1.15	2.09	4	0.47
		F_2_	*qNPF9-F_2_*	9	122.46	TP_46766	TP_46851	TP_46851	2.34	9.16	3.9	0.81
			*qNPF14-F_2_*	14	66.48	TP_67968	TP_68232	TP_67968	1.88	12.03	3.1	0.91
2	Days to first pistillate flower appearance	F_2:3_	*qDPF3-F_3_*	3	50.59	TP_9388	TP_8845	TP_8845	1.41	0.05	36.1	-0.42
			*qDPF5a-F_3_*	5	71.66	TP_11213	TP_11334	TP_11213	1.96	15.13	3.2	-7.25
			*qDPF5b-F_3_*	5	72.16	TP_11213	TP_11334	TP_11213	2.46	15.33	3.3	-7.30
			*qDPF14-F_3_*	14	53.16	TP_67682	TP_67839	TP_67682	0.06	17.34	4.4	7.92
			*qDPF16a-F_3_*	16	74.12	TP_74581	TP_74591	TP_74591	7.78	58.75	35.8	-19.22
			*qDPF16b-F_3_*	16	74.62	TP_74581	TP_74591	TP_74591	7.28	58.75	35.9	-19.22
		F_2_	*qDPF3a-F_2_*	3	66.43	TP_6352	TP_6283	TP_6283	0.17	9.22	2.4	-2.77
			*qDPF3b-F_2_*	3	66.6	TP_6352	TP_6283	TP_6283	0.00	8.64	2.5	-2.66
3	Sex ratio (male: female)	F_2:3_	*qSR9a-F_3_*	9	5.01	TP_46003	TP_46763	TP_46763	3.59	7.92	2.5	-1.78
			*qSR9b-F_3_*	9	5.51	TP_46003	TP_46763	TP_46763	3.09	7.62	2.6	-1.75
			*qSR13-F_3_*	13	55.77	TP_59825	TP_59813	TP_59813	1.23	11.51	3.9	-2.23
			*qSR14a-F_3_*	14	68.88	TP_68232	TP_68500	TP_68232	0.48	16.24	6.3	2.51
			*qSR14b-F_3_*	14	69.38	TP_68232	TP_68500	TP_68500	0.12	16.90	6.4	2.56
		F_2_	*qSR14a-F_2_*	14	63.52	TP_68643	TP_67968	TP_67968	1.08	7.60	6.2	1.58
			*qSR14b-F_2_*	14	64.02	TP_68643	TP_67968	TP_67968	0.58	20.95	6.3	2.65
			*qSR16a-F_2_*	16	23.74	TP_71910	TP_72004	TP_72004	0.36	6.70	2.5	-1.62
			*qSR16b-F_2_*	16	24.16	TP_72004	TP_72073	TP_72004	0.06	6.35	2.6	-1.58

*^∗^QTL named as qxxxya-F_3_ or F_2_, with “xxx” being the trait abbreviation, ‘y’ the number of the linkage group, ‘a’ the letter to specify different QTLs for the same trait in same linkage group and ‘F_2_ or F_3_’ the generation (F_2_ or F_3_) in which the trait was phenotyped. ^#^Additive effect was positive when the “DBGy-201” allele increased the trait score and was negative when the “Pusa Do Mousami” allele increased trait score*.

### Sex Ratio (♂: ♀)

Four major QTLs were identified for sex ratio (♂: ♀), one on LG-13 and three on the linkage group LG-14. The QTL qSR13-F_3_ was located between flanking markers TP_59825 and TP_59813 on LG-13, had shown LOD value of 3.90 and explaining 11.51% of phenotyping variation (*R*^2^%). The QTLs *qSR14a-F_3_* and *qSR14b-F_3_* were located between flanking markers TP_68232 and TP_68500 on LG-14, had shown LOD value of 6.30 and 6.40, respectively (*R*^2^ = 16.24 and 16.90%, respectively). The *qSR14b-F_2_* was located between flanking markers TP_68643 and TP_67968 on LG-14 with LOD value of 6.30 (*R*^2^ = 20.95%). All four major QTLs together explained 65.60% of phenotyping variation. The QTLs *qSR14a-F_3_, qSR14b-F_3_*, and *qSR14b-F_2_* showed positive additive effect, indicating allele for increasing sex ratio was contributed by DBGy-201, whereas QTL *qSR13-F_3_* showed negative additive effect, indicating allele for increasing sex ratio was contributed by Pusa Do Mousami.

### Node at First Pistillate Flower Appearance

Two major QTLs were identified for node at first pistillate flower appearance one each on the linkage group LG-9 and LG-14. The *qNPF9-F_3_* was located between flanking markers TP_46756 and TP_46766 on LG-9, showing LOD value of 4.00 (*R*^2^ = 13.94%). The *qNPF14-F_2_* was located between flanking markers TP_67968 and TP_68232 on LG14 (*R*^2^ = 12.03%). Both the QTLs together contributed 25.97% of phenotyping variation and showed positive additive effect indicating positive allele for increasing node at first pistillate flower appearance was contributed by female parent PVGy-201.

### Days to First Pistillate Flower Appearance

Economically most important horticultural traits in bitter gourd for getting early and high yield are early flowering and sex ratio which are highly variable, affected by genetic, environmental and hormonal factors. Five major additive QTLs were identified for days to first pistillate flower appearance, two each on the linkage groups LG-5 and LG-16; and one on LG-14. The QTLs *qDPF5a-F_3_* and *qDPF5b-F_3_* were located between flanking markers TP_11213 and TP_11334 on LG-5, showed LOD value of 3.20 and 3.30 explaining 15.13 and 15.33% of phenotyping variation (*R*^2^%), respectively. The QTLs *qDPF16a-F_3_* and *qDPF16b-F_3_* were located between flanking markers TP_74581 and TP_74591 on LG-16 had shown LOD value of 35.80 and 35.90, respectively (*R*^2^ = 58.75% individually). The *qDPF14-F_3_* was located between flanking markers TP_67682 and TP_67839 on LG-14 with LOD value of 4.40 and explaining 17.34% of phenotyping variation (*R*^2^%) and both QTLs explained 25.97% of phenotyping variation. The QTL *qDPF14-F_3_* showed positive additive effect, indicating allele for increasing days to first pistillate flower appearance was contributed by PVGy-201, whereas QTLs *qDPF5a-F_3_, qDPF5b-F_3_, qDPF16a-F_3_*, and *qDPF16b-F_3_* showed negative additive effect, indicating allele for increasing days to first pistillate flower appearance was contributed by Pusa Do Mousami.

## Discussion

To date, there is no precise report on QTL mapping for any trait in bitter gourd and only few studies have been reported. Kole et al. (2012) mapped five of each qualitative and five quantitative trait loci by using AFLP markers; Wang and Xiang (2013) did the genetic linkage map for 13 horticulture traits by RAD-seq analysis; Matsumura et al. (2014) identified one SNP marker, GTFL-1 linked to the gynoecious locus at a distance of 5.46 cM and Cui et al. (2018) identified SNP markers relate to sex expression, fruit epidermal structure and fruit color. In our present study, we applied high throughput GBS technology with type-II restriction endonuclease *ApeKI* (GCWGC) (Elshire et al., 2011) to identify SNPs in F_2_ and F_2:3_ segregated populations for economically important traits like, sex and earliness in bitter gourd. The draft genome of bitter gourd not available in public domain, we performed non-reference based GBS with UNEAK pipeline (Lu et al., 2013) and a total 2013 SNP markers used to construct 20 LGs spanned over 2329.2 cM. GBS technology in the present study provided the high mean marker density genetic map 0.86 (marker/cM), which higher than the 0.30 mean marker density reported by Matsumura et al. (2014), 0.42 by Urasaki et al. (2017), and 0.46 by Cui et al. (2018). Our genetic map had an excess of LGs (20) relative to the haploid chromosome number (*n* = 11) (Bharathi et al., 2011) even though a significant number of markers (2013 SNPs) were binned to genetic map. The failure to obtain the basic chromosome number was likely due to a relatively small F_2_ population size (Silva et al., 2007) and type of mapping population. To address these problems, it will be necessary to increase the size of mapping population or increase the marker density further or replace the mapping population with RILs.

Inheritance pattern of qualitative traits were investigated in bitter gourd cross DBGy-201 × Pusa Do Mousami of F_2_ population revealed that, the gynoecious trait (*gy-1*) single recessive gene (Ram et al., 2006; Behera et al., 2009; Matsumura et al., 2014; Mishra et al., 2015), in contrary two pairs of genes reported by Cui et al. (2018). We succeeded in finding a SNP marker for most important trait in bitter gourd, i.e., gynoecy (*gy-1*), marker TP_54890 was very close to *gy-1* gene, with a distance of 3.04 cM. Gynoecy (*gy-1*) trait useful for low cost and quality hybrid seed production, early and high yielding hybrids. The *gy-1* gene flanked to markers TP_54865 and TP_54890 on LG 12 are extremely useful in marker development and MAS for rapid development of various gynoecious lines with different genetic background of best combiner for development of early and high yielding hybrids. Based on a RAD-seq analysis of F_2_ progeny Matsumura et al. (2014) identified one SNP marker, GTFL-1 linked to the gynoecious locus at a distance of 5.46 cM and similarly Cui et al. (2018) located the two QTLs *gy1.1* and *gy1.2* at distal end of linkage map by RAD-based genetic map for anchoring scaffold sequences in bitter gourd. We identified two SNPs within 5 cM distance from *gy-1* gene, TP_54890 (3.04 cM) and TP_54923 (4.14 cM). In this study we have taken highly diversified parents with highly segregated mapping population and less base recognition restriction endonuclease *Ape*KI (GCWGC) and constructed linkage map with high number (2013) quality SNPs.

The three quantitative traits were mapped with 22 QTLs were identified using CIM. Out of 22 QTLs, 13 QTLs were derived from the commercial variety Pusa Do Mousami those showed negative additive effect and 9 QTLs were derived from PVGy-201 showed positive additive effect. Earliness is an important trait for realizing the potential economic yield in as less time as possible which is an important consideration for a vegetable grower in bitter gourd (Behera et al., 2010). Earliness in bitter gourd is attributed to node and days to first pistillate flower appearance and sex ratio. One of the most interesting thing in frequency distribution of F_2_ and F_2:3_ populations in bitter gourd was bimodal distribution of sex ratio (♂: ♀) which revealed that sex ratio is governed by semi-quantitative genes and some major genes may be modified by other minor genes (Matsumura et al., 2014). The inheritance of sex ratio (♂: ♀), node and days to first pistillate flower appearance, among monoecious F_2_ individuals and F_2:3_ families revealed that more tendency of pistillate flower might have a semi-dominant effect on the sex ratio or that additional genes around the *gy-1* locus might be responsible for the determination of the sex ratio (Matsumura et al., 2014) and predominance of major genes toward lower node and days to first pistillate flower appearance.

In bitter gourd, yield may be increased by altering plant architecture to produce gynoecious, early flowering (node and days to first pistillate flower appearance) and cultivars with better sex ratio. In the present study we identified QTLs that explain significant portions of the observed phenotypic variation for plant architecture. The appearance of the first pistillate flower at a lower node number is an indication of the earliness of the variety (Trivedi, 1983). On the other hand, the higher the node number for the position of the first pistillate flower, the greater would be the production of pistillate flowers, and a shorter interval between the appearance of the first staminate flower and the first pistillate flower indicates shorter plant life (Mini Raj et al., 1993). Manipulation of sex expression can influence fruit quality, yield, cropping methods, and breeding strategies. Similarly, gynoecy in bitter gourd has been associated with earlier fruit production and higher yield (Behera et al., 2009).

Two major QTLs together with 25.97% phenotyping variation for node to first pistillate flower appearance and both QTLs *qNPF9-F_3_* and *qNPF14-F_2_* showed positive additive effects contributed from PVGy-201 alleles increased the nodes for first pistillate flower appearance at 1.24 and 0.91 node number, respectively. Similarly, Wang and Xiang (2013) identified three QTLs (fffn4.1, fff5.1, and fffn9.1) two QTLs in two different locations by Cui et al. (2018) in bitter gourd. Three (Fazio et al., 2003) and six (Yuan et al., 2008) QTLs in cucumber and nine QTLs based on an interspecific genetic map of *Luffa* (Cui et al., 2015) for node to first pistillate flower appearance.

Three major QTLs (*qDPF14-F_3_, qDPF5a-F_3_*, and *qDPF5b-F_3_*) together explained 47.80% of phenotyping variation and other two major QTLs (*qDPF16a-F_3_* and *qDPF16b-F_3_*) explained 58.75% of phenotyping variation individually for days to first pistillate flower appearance. The QTL *qDPF14-F_3_* showed positive additive effect, thus PVGy-201 alleles for increased 7.92 days to first pistillate flower appearance, whereas QTLs *qDPF5a-F_3_, qDPF5b-F_3_, qDPF16a-F_3_*, and *qDPF16b-F_3_* showed negative additive effect, thus Pusa Do Mousami alleles increased days to first pistillate flower appearance by 7.25, 7.30, 19.22, and 19.22 days, respectively. Similarly four QTLs mapped for days to anthesis in cucumber (Fazio et al., 2003). Two QTLs were identified in zucchini based on GBS technology (Pau et al., 2017) for days to first female pistillate flower appearance.

Four major QTLs together explained 65.60% of phenotyping variation for sex ratio. The QTLs *qSR14a-F_3_, qSR14b-F_3_*, and *qSR14b-F_2_* showed positive additive effects, thus DBGy-201 alleles from these QTLs increased the sex ratio by 2.51, 2.56, and 2.65%, respectively, whereas QTL *qSR13-F_3_* showed negative additive effect, thus Pusa Do Mousami alleles from this QTL increased the sex ratio by 2.23%. Similarly three QTLs were identified each in cucumber (Yuan et al., 2008) and bitter gourd (Wang and Xiang, 2013) for sex ratio.

## Conclusion

A total of 22 QTLs for three traits were identified and mapped on 20 LGs and the gynoecy (*gy-1*) was mapped with SNP marker TP_54890 with a distance of 3.04 cM on LG-12. Major QTLs were mapped in present study has the potential to significantly improve the efficiency of MAS for gynoecious gene, sex ratio and earliness for development of best hybrids or varieties.

## Author Contributions

PGR and TKB performed and conceived the experiments, collected data, writer and analyst of results. TKB, AG, and ADM supervised the project. GSJ and GB management and technical support. All authors reviewed and approved this submission.

## Conflict of Interest Statement

The authors declare that the research was conducted in the absence of any commercial or financial relationships that could be construed as a potential conflict of interest.

## Footnotes

1 http://sourceforge.net/projects/tassel/
