# Supplementary material for: Mapping and QTL Analysis of Gynoecy and Earliness in Bitter Gourd (Momordica charantia L.) Using Genotyping-by-Sequencing (GBS) Technology
Source: Front Plant Sci. 2018 Oct 31;9:1555. doi: 10.3389/fpls.2018.01555 (PMC6220052; doi:10.3389/fpls.2018.01555)
Supplement: Supplementary file 2 [file Presentation_1.PPTX]

## Slide 1
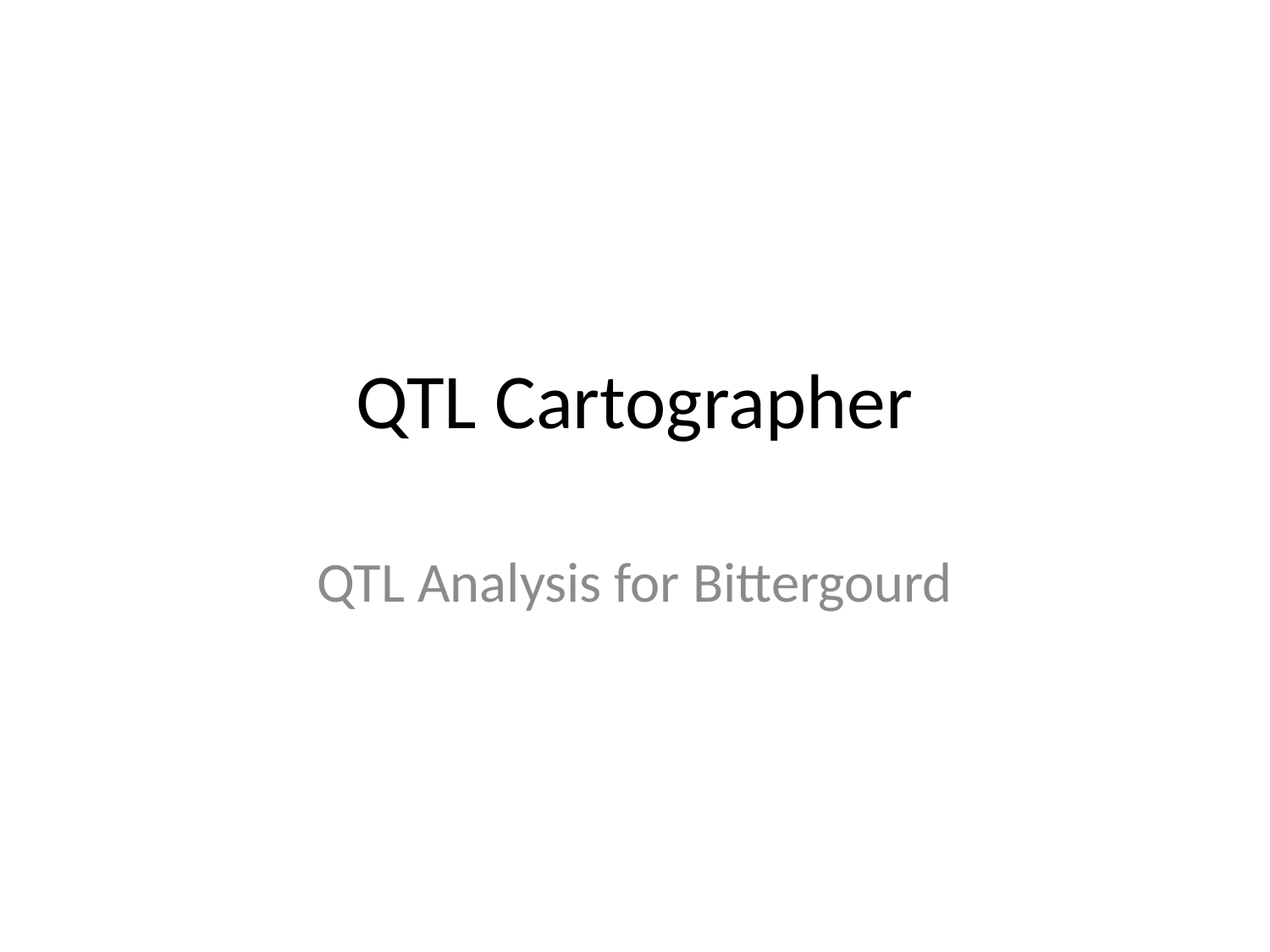

# QTL Cartographer
QTL Analysis for Bittergourd

## Slide 2
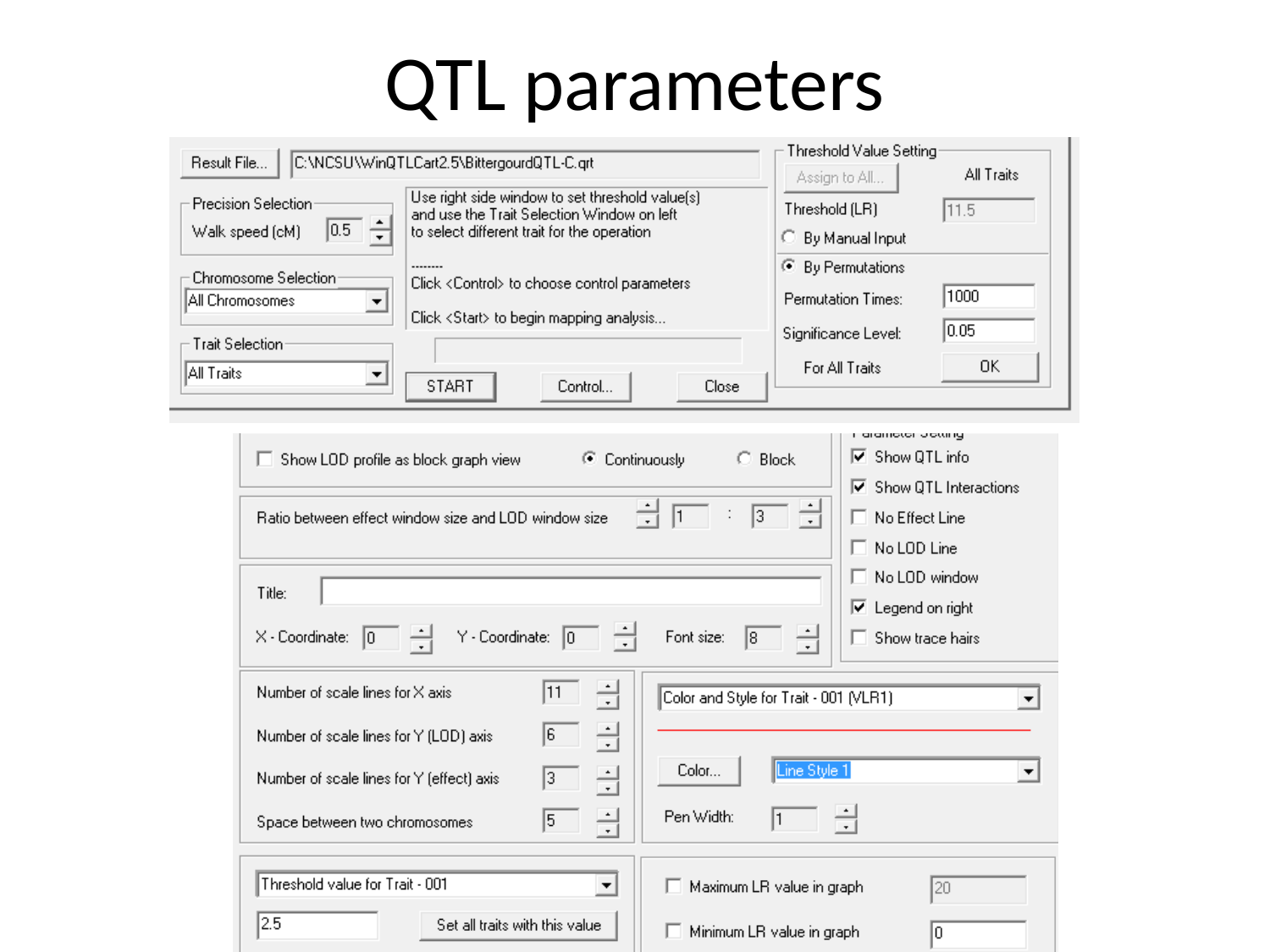

# QTL parameters

## Slide 3
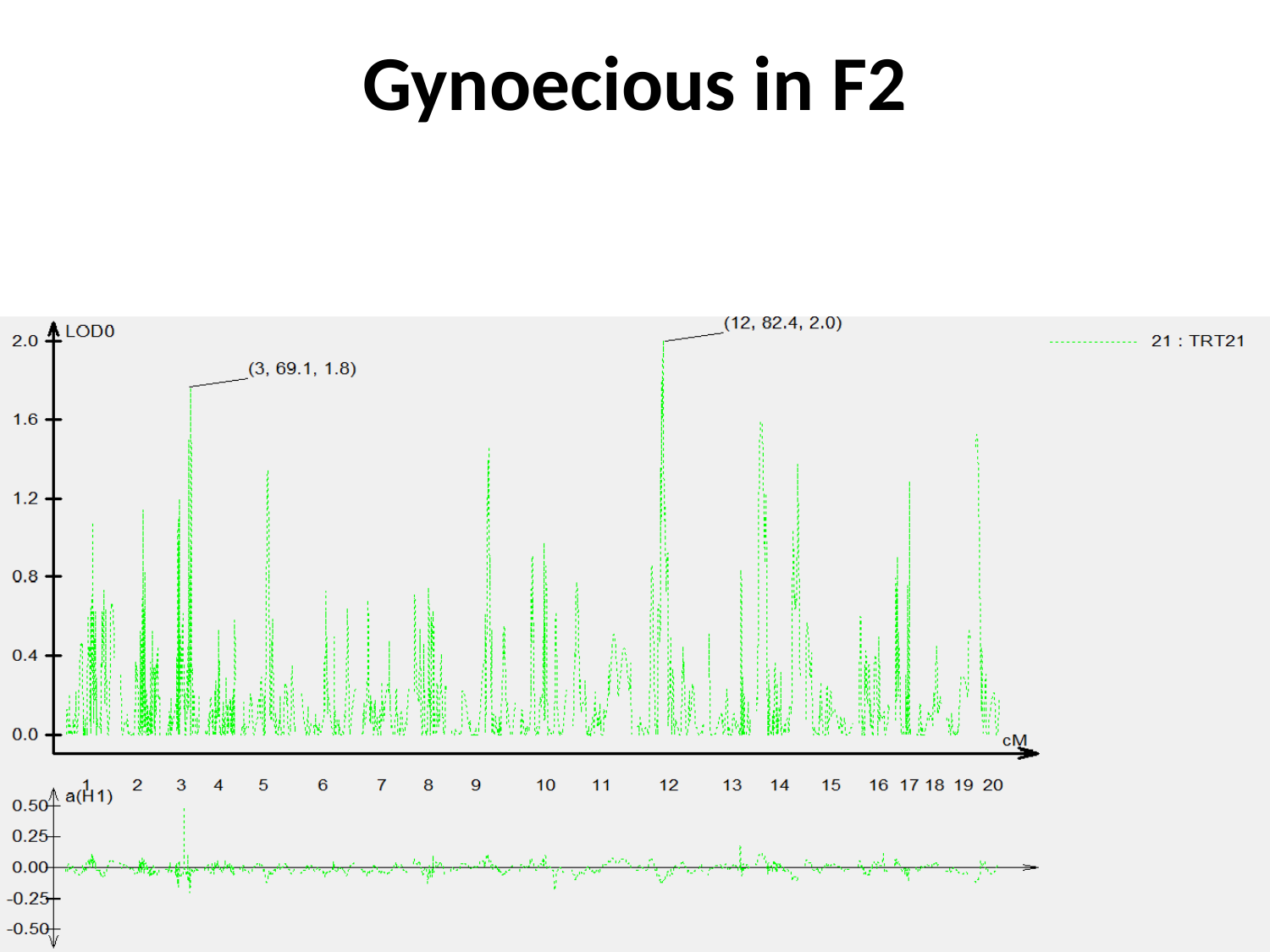

Gynoecious in F2

## Slide 4
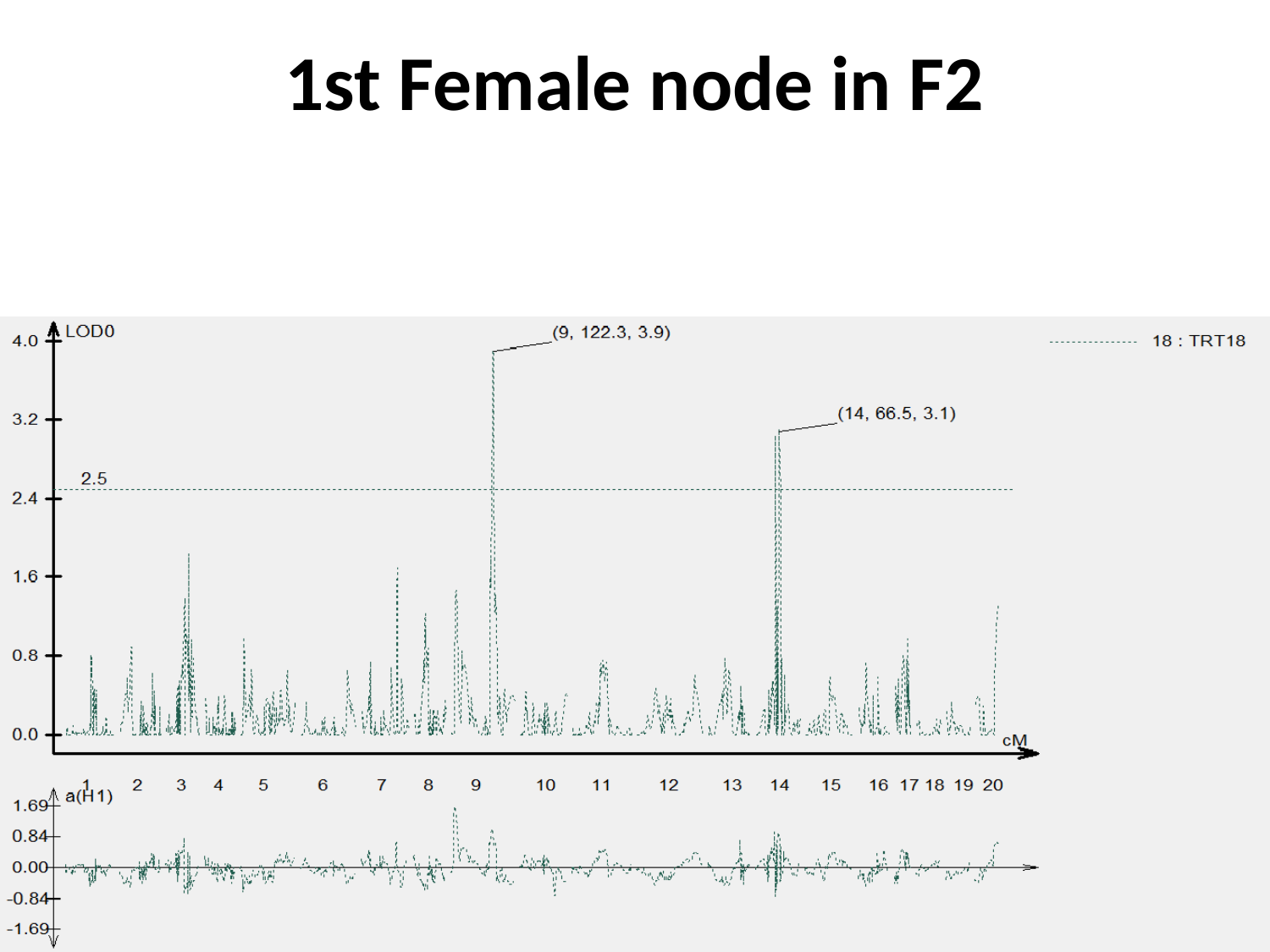

1st Female node in F2

## Slide 5
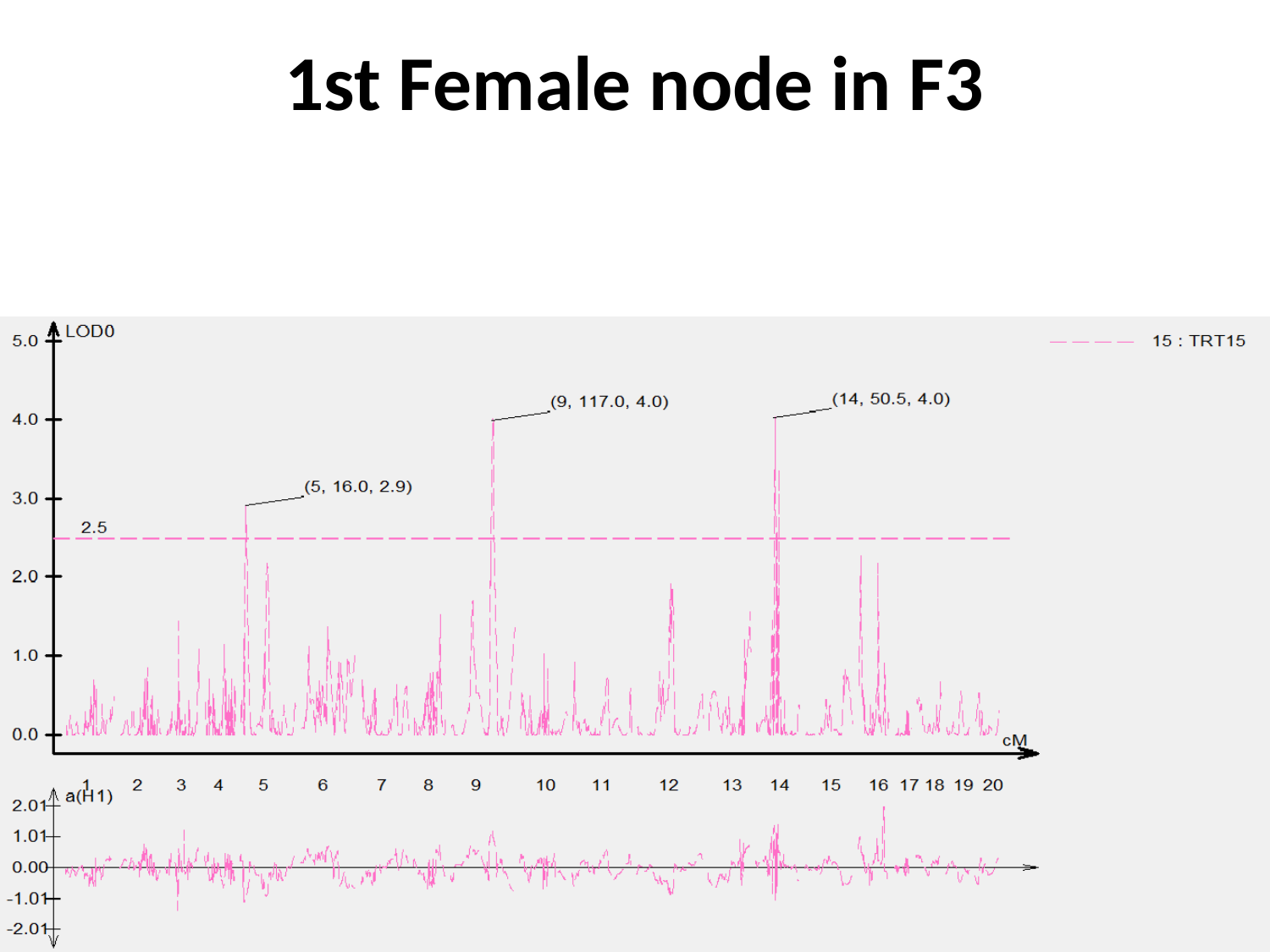

1st Female node in F3

## Slide 6
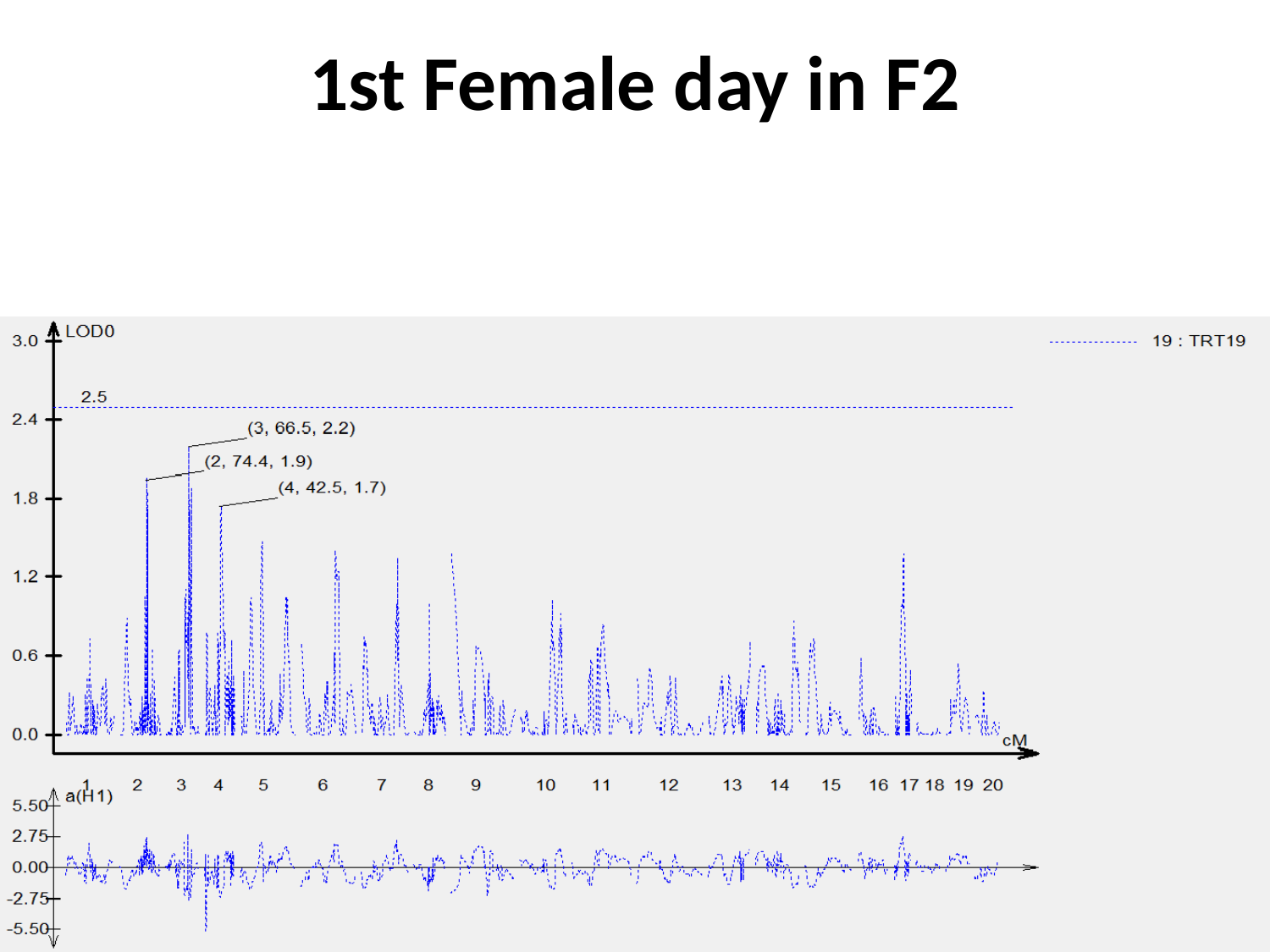

1st Female day in F2

## Slide 7
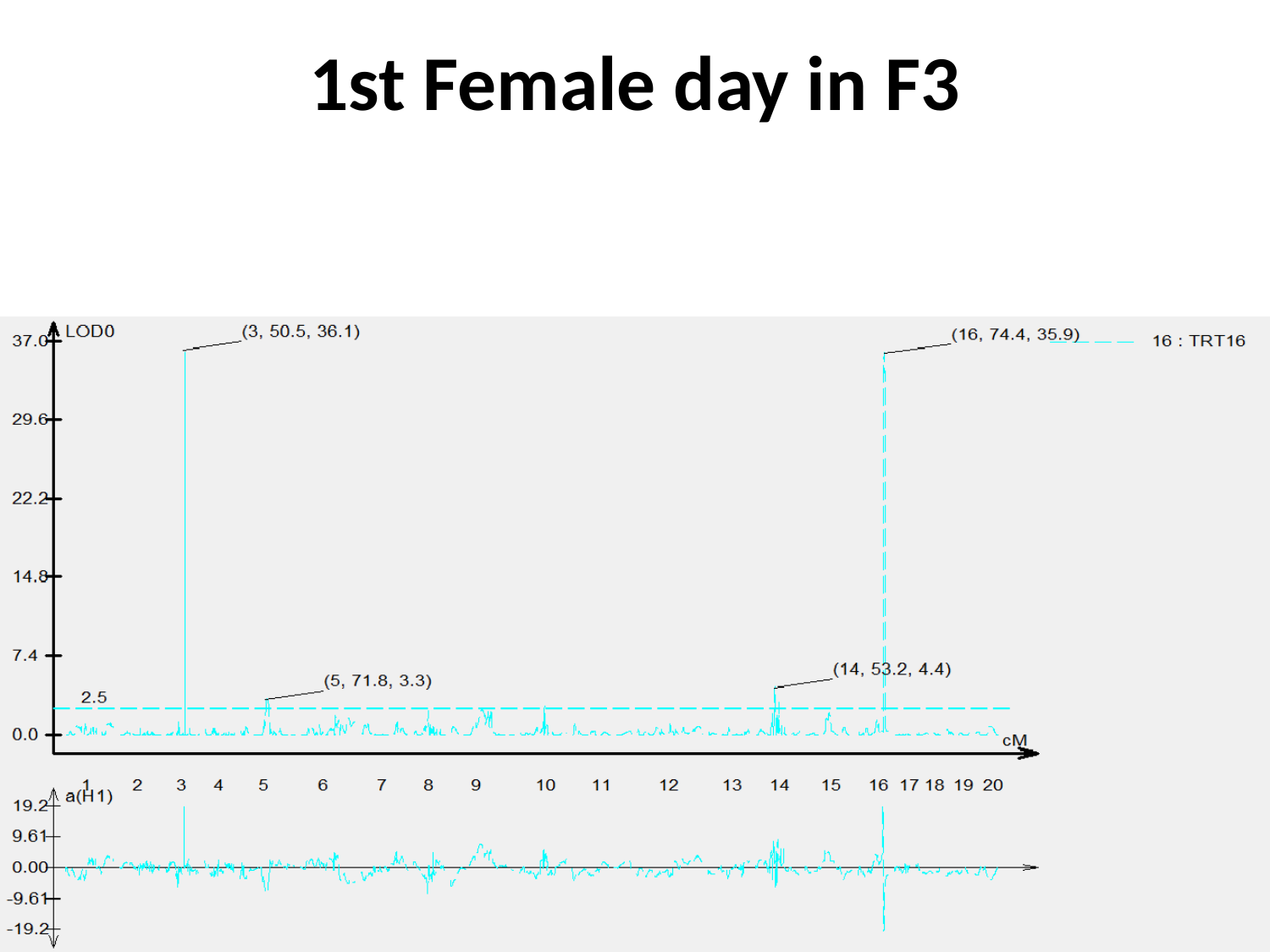

1st Female day in F3

## Slide 8
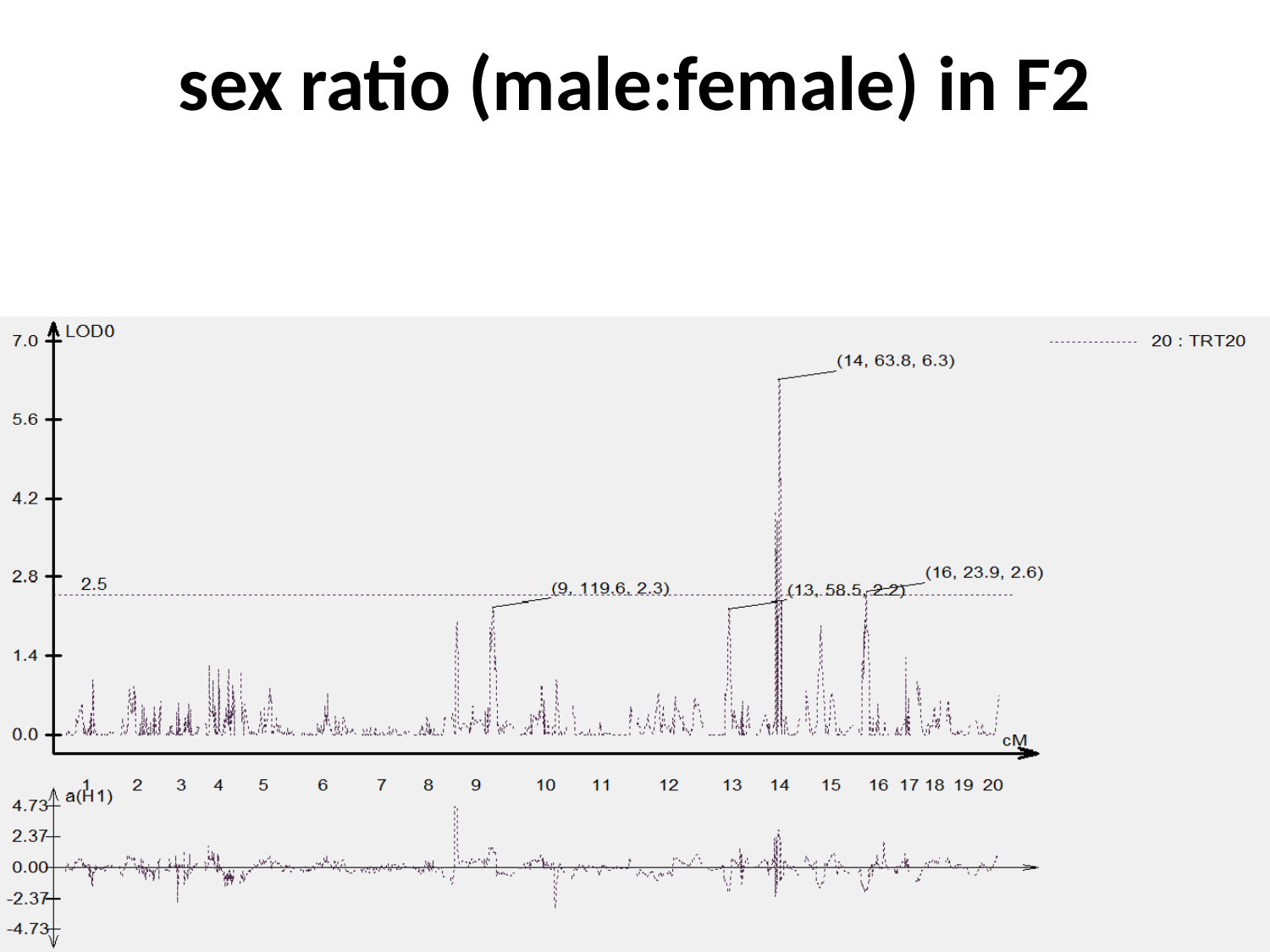

sex ratio (male:female) in F2

## Slide 9
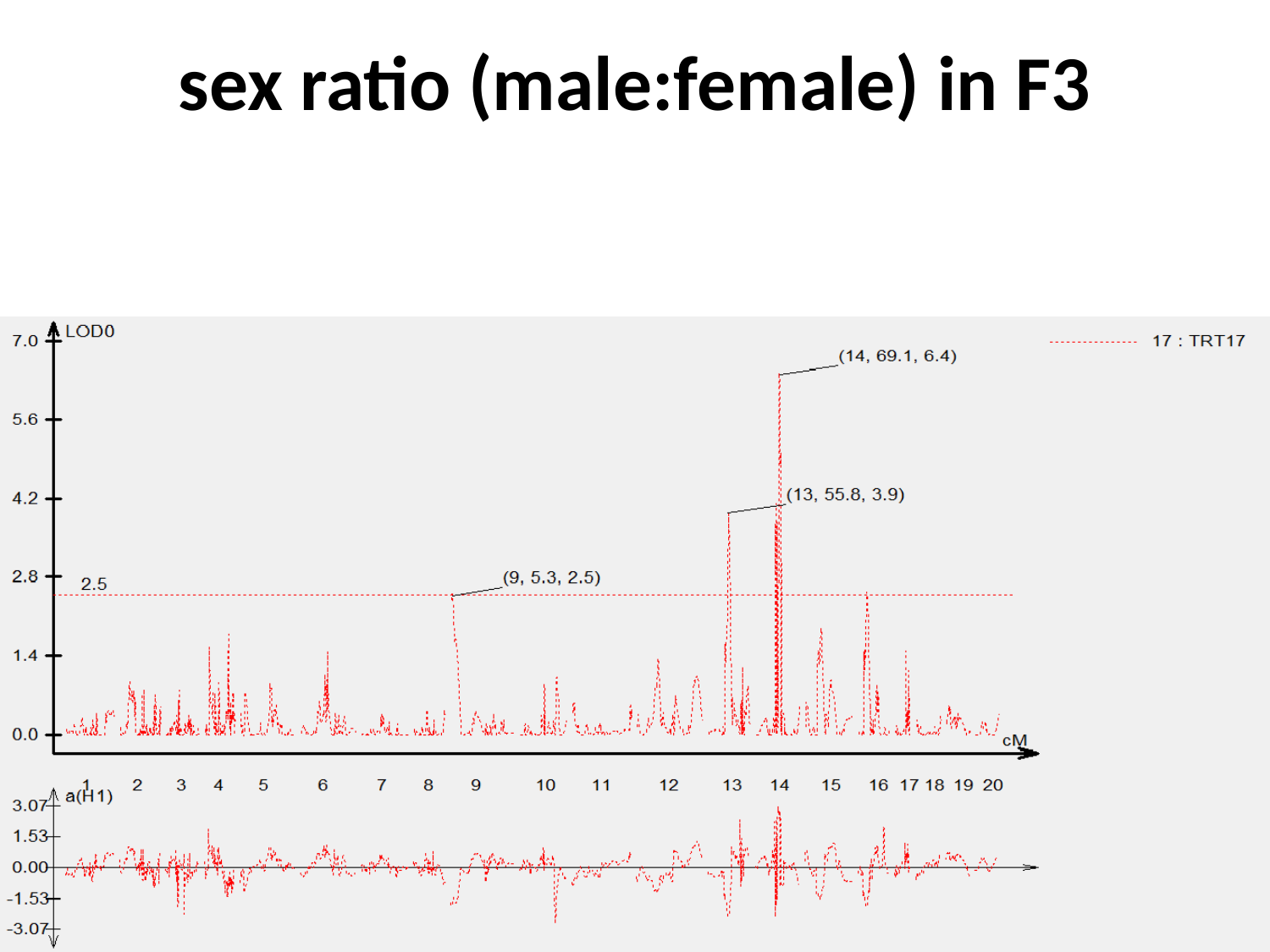

sex ratio (male:female) in F3
