# Supplementary material for: Mapping and QTL Analysis of Gynoecy and Earliness in Bitter Gourd (Momordica charantia L.) Using Genotyping-by-Sequencing (GBS) Technology
Source: Front Plant Sci. 2018 Oct 31;9:1555. doi: 10.3389/fpls.2018.01555 (PMC6220052; doi:10.3389/fpls.2018.01555)
Supplement: Supplementary file 3 [file Data_Sheet_1.DOCX]

**Choosing most suitable restriction enzyme (RE) and optimization**

To perform SNP genotyping on Bittergourd 93 samples including parental genotypes using Gentotyping By Sequencing method (GBS); sequencing for generating Linkage map using JOINMAP v4.1 & QTL analysis using QTL cartographer v2.5. Choosing most suitable restriction enzyme is most important for success of sequencing.

ApekI Enzyme will be used for library preparation. Preparing **one set of 94 Plex** library (93 lines+blank). Generating sequencing RAW data for the optimized GBS library using Illumina True Seq V4 Chemistry.

**Bioinformatics Analysis includes**:

- Filtering the high quality reads from the RAW data.
- Assembly of high quality filtered reads (Analysis using UNEAK pipeline from TASSEL as the reference genome is not available).
- Mining out polymorphic SNP markers from the assembled data.

**Sample Information**:

- 93 Accessions
- Negative Control (BLANK)
- **ApeKI Enzyme Library:** 93 SAMPLES +Blank (refer to the plate format given below)

| **BITTERGOURD PLATE MAP** | | | | | | | | | | | | |
| --- | --- | --- | --- | --- | --- | --- | --- | --- | --- | --- | --- | --- |
|  | **1** | **2** | **3** | **4** | **5** | **6** | **7** | **8** | **9** | **10** | **11** | **12** |
| **A** | 1 | 2 | 3 | 4 | 5 | 6 | 7 | 8 | 9 | 10 | 11 | 12 |
| **B** | 13 | 14 | 15 | 16 | 17 | 18 | 19 | 20 | 21 | 22 | 23 | 24 |
| **C** | 25 | 26 | 27 | 28 | 29 | 30 | 31 | 32 | 33 | 34 | 35 | 36 |
| **D** | 37 | 38 | 39 | 40 | 41 | 42 | 43 | 44 | 45 | 46 | 47 | 48 |
| **E** | 49 | 50 | 51 | 52 | 53 | 54 | 55 | 56 | 57 | 58 | 59 | 60 |
| **F** | 61 | 62 | 63 | 64 | 65 | 66 | 67 | 68 | 69 | 70 | 71 | 72 |
| **G** | 73 | 74 | 75 | 76 | 77 | 78 | 79 | 80 | 81 | 82 | 83 | 84 |
| **H** | 85 | 86 | 87 | 88 | 89 | 90 | 91 | 92 | 93 |  |  |  |

**Restriction Enzyme Genome Optimization:**


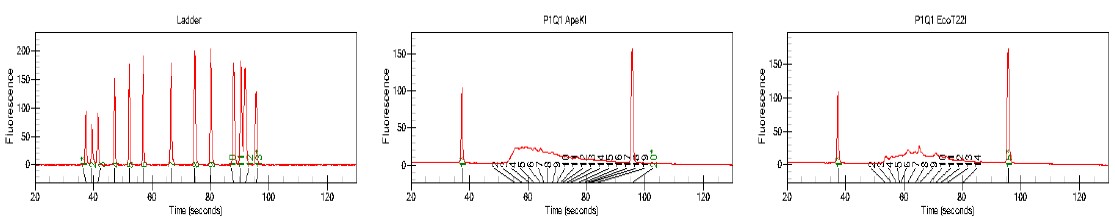

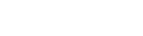

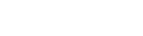


ApeKI

EcoT22I


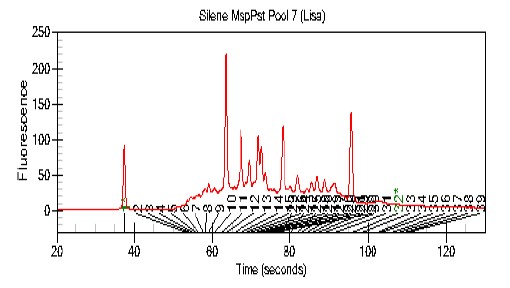

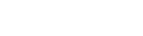

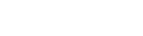


MspI/PstI

PstI


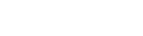

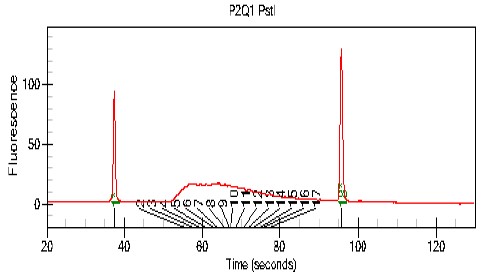

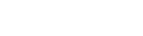


***Note: Best Library fragment distribution was identified using ApekI Enzyme. Hence ApekI was chosen for library preparations for all Bitter gourd Accessions.***

**Optimized Library details:**

- **Single** End adapters were used for library preparation.
- For preparing each library we have used 94 different barcodes for tagging samples. These barcodes are of variable length from 5-10 nt in lengths.

**Adaptors sequences for *ApeKI***

**The ApeKI barcode adapters:**

5′-ACACTCTTTCCCTACACGACGCTCTTCCGATCTxxxx and

5′-CWGyyyyAGATCGGAAGAGCGTCGTGTAGGGAAAGAGTGT, where “xxxx” and

“yyyy” denote the barcode and barcode complement, respectively.

**The ApeKI common adapter**:

5′-CWGAGATCGGAAGAGCGGTTCAGCAGGAATGCCGAG and

5′-CTCGGCATTCCTGCTGAACCGCTCTTCCGATCT

**Final Bitter gourd Library QC Results on Agilent’s Bioanlyzer 2100:**


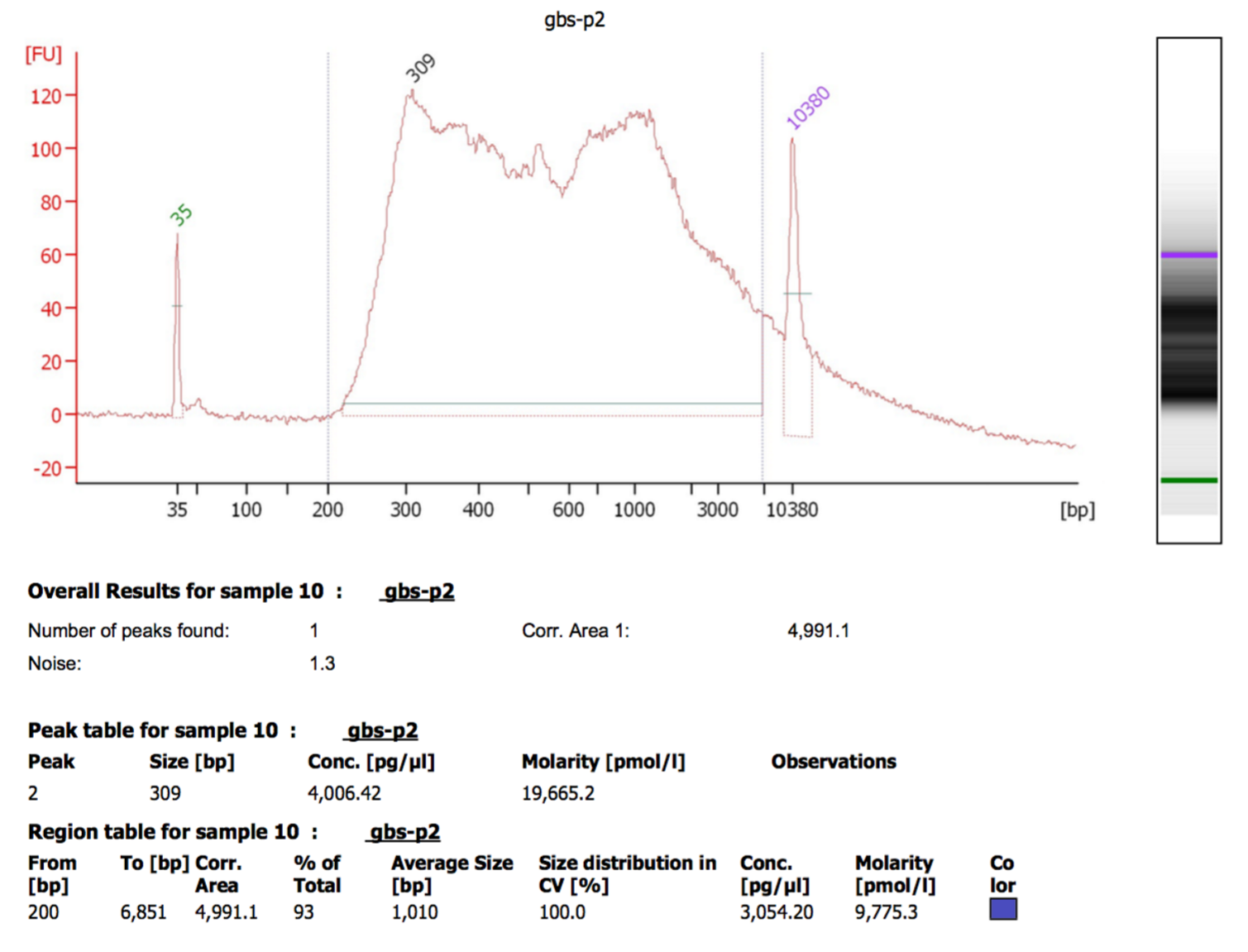


**Summarised Details for Final Libraries:**

- 94 well plate generated good ApeKI Enzyme Libraries which have passed the QC criteria.
- There are no adapter dimers and the library fragments are well within the sequencing size range of the Illumina system, for Sequencing using Illumina True Seq Version 4 Chemistry.
