# Supplementary material for: Mapping and QTL Analysis of Gynoecy and Earliness in Bitter Gourd (Momordica charantia L.) Using Genotyping-by-Sequencing (GBS) Technology
Source: Front Plant Sci. 2018 Oct 31;9:1555. doi: 10.3389/fpls.2018.01555 (PMC6220052; doi:10.3389/fpls.2018.01555)

**Detailed Library Preparation Protocol followed for GBS:**

1. **Preparing Single Stranded Adapters**

Resuspend lyophilized oligos in TE to 200uM. (Multiply the *n* moles by 5 to get the volume of TE you need to use).

1. **Annealing Adapters (Top and Bottom Oligo to make double stranded adapters):**
   1. In a PCR plate bring together the barcoded adapters.
   2. In a 0.2 ml tube bring together the common adapter.

Top strand DNA oligo (200 μM): 25ul

Bottom strand DNA oligo (200 μM): 25ul

TE 50ul

Total volume 100ul

Annealed Molarity: 50mM

**In Thermocycler:**

- 95 degrees for 2 minutes.
- Ramp to 25 degrees by 0.1 degree per second.
- Hold at 25 degrees for 30 minutes.
- Hold at 4 degrees forever.

1. **First Dilution:**
2. Bring together barcoded adapters in 1.1 ml microdilution tubes:5.6ul of annealed barcoded adapters.
3. 995ul of 1X TE.
4. Vortex and spin down.
5. Total Volume: 1,000 ul.

Bring together common adapter in 1.5ml centrifuge tube:

- All of the annealed common adapter (100 ul).
- 900 ul of 1X TE.
- Vortex and spin down.
- Total Volume: 1,000 ul.

1. **Adapter Quantification:**

Quantify the barcoded and common adapters using PicoGreen.

1. **Concentrated Stock:**

The adapters work in pairs for the PCR step in the protocol. One must have each of them in the ligation for it to proceed properly. The second (or common) adapter is the same in each ligation. Mix the barcoded adapter with the common adapter to get the concentrated stock. Bring together in a PCR plate:

- 300 ng barcoded adapter.
- 300 ng common adapter.
- Add 1x TE to make 200 ul.
- Vortex, spin.
- The concentrated stock is at 3ng/ul.

1. **Working Adapter Stock:**

- Dilute the above stock mix 1:4 stock: water.
- The working adapter stock is at 0.6ng/ul.
- For each species the optimal amount must be determined experimentally.

**Genome Optimizations:**

1. **gDNA QC & Enzyme Selection:**
   - 1. Restriction enzymes which are methylation-sensitive (do not cut frequently in the major repetitive fraction of the genome) and produce overhangs (“sticky ends”) are the best choice.
     2. Test restriction digestions reactions should be set up to check whether DNA extractions are of sufficient quality for library construction, run an agarose gel with aliquotes of cut and uncut sample DNA.
2. **Adapter titration**
   1. The proper ratio of adapters to sample DNA “sticky” ends is critical to the success of experiment.
   2. Less adapters encourages chimera formation among sample DNA molecules.
   3. Abundant results in formation of adapter dimmers & produce DNA sequence data, thus wasting reagents and machine time.
   4. A range of Adapter ratio can be used for titration against a set amount of digested DNA to empirically determine the correct ratio of adapters to sample DNA ends.
   5. This is required only once for a species. This titration needs to be performed for each restriction enzyme used.
   6. The following protocol uses eight different adapter concentrations.
3. **Restriction Digestion Set up:**

In an 8 strip tube set up each of the 8 reactions as follows:

| **Reagent** | **Volume (µL)** |
| --- | --- |
| Genomic DNA (100ng / µL) | 2 |
| NEB Buffer 3 (10X) | 2 |
| *Ape*KI (4U/µL) | 1 |
| dH_2_0 | 15 |
| Total | 20 |

Tap to mix, Spin briefly in tabletop centrifuge.Incubate at 75°C for 2 hours.

1. **Ligate varying amounts of adapters**

| **Reagent** | **Tube1 (µL)** | **Tube 2(µL)** | **Tube 3 (µL)** | **Tube 4 (µL)** | **Tube 5 (µL)** | **Tube 6 (µL)** | **Tube 7 (µL)** | **Tube 8 (µL)** |
| --- | --- | --- | --- | --- | --- | --- | --- | --- |
| Digested DNA | 20 | 20 | 20 | 20 | 20 | 20 | 20 | 20 |
| NEB Ligase buffer (10X) | 5 | 5 | 5 | 5 | 5 | 5 | 5 | 5 |
| Adapter mix* (0.3ng/ µL each adapter) | 6 | 8 | 12 | 14 | 16 | 18 | 20 | 24 |
| dH2O | 18 | 16 | 12 | 10 | 8 | 6 | 4 | 0 |
| NEB T4 DNA ligase (400 CELU*/ µL) | 1 | 1 | 1 | 1 | 1 | 1 | 1 | 1 |
| Total | 50 | 50 | 50 | 50 | 50 | 50 | 50 | 50 |

**Note:** Use only one set of adapter pairs (*i.e*., one of the 96 barcode adapters with a barcode listed in Table S1 along with the common adapter).

- - Add the ligase to the side of the tube so that it is added to all the reaction tubes at the same time.
  - Spin down briefly in tabletop centrifuge.
  - Incubate at 22°C for 60 minutes.
  - Deactivate the ligase by incubating at 65°C for 30 minutes. Cool to 4°C.

1. **Clean-up**
   - Cleanup with Qiagen PCR cleanup kit per kit instructions.
   - Elute in 50µl elution buffer (EB).
2. **PCR Amplification**

For each of the 8 reactions from above, set up a PCR reaction as follows:

| **Reagent** | **Volume (µL)** |
| --- | --- |
| DNA from previous step | 10 |
| NEB 2x Taq Master Mix | 25 |
| PCR Primer Mix (12.5 pmol/µL each primer) | 2 |
| dH_2_O | 13 |
| **Total** | **50** |

- Amplify using the following PCR cycling protocol:

1. 5 minutes at 72°C
2. 30 seconds at 98°C
3. 18 cycles of:
   - 10 seconds at 98°C
   - 30 seconds at 65°C
   - 30 seconds at 72°C
4. 5 minutes at 72°C
5. Hold at 4°C.

- For each of the PCR reactions, purify with Qiagen PCR cleanup kit per kit instructions.
- Elute in 30µL Qiagen EB.

**7. Evaluate libraries**

Figure below shows output from the Bio-Rad Experion® for a library made with an excess of adapters. Note that a large adapter dimer peak is evident around 128 bp. The very small peak around 70 bp consists of PCR primer dimers. Run each of the 8 samples on the Experion® according to manufacturer’s instructions. Determine the amount of adapter that produces a good library but shows **no adapter dimer peak.**


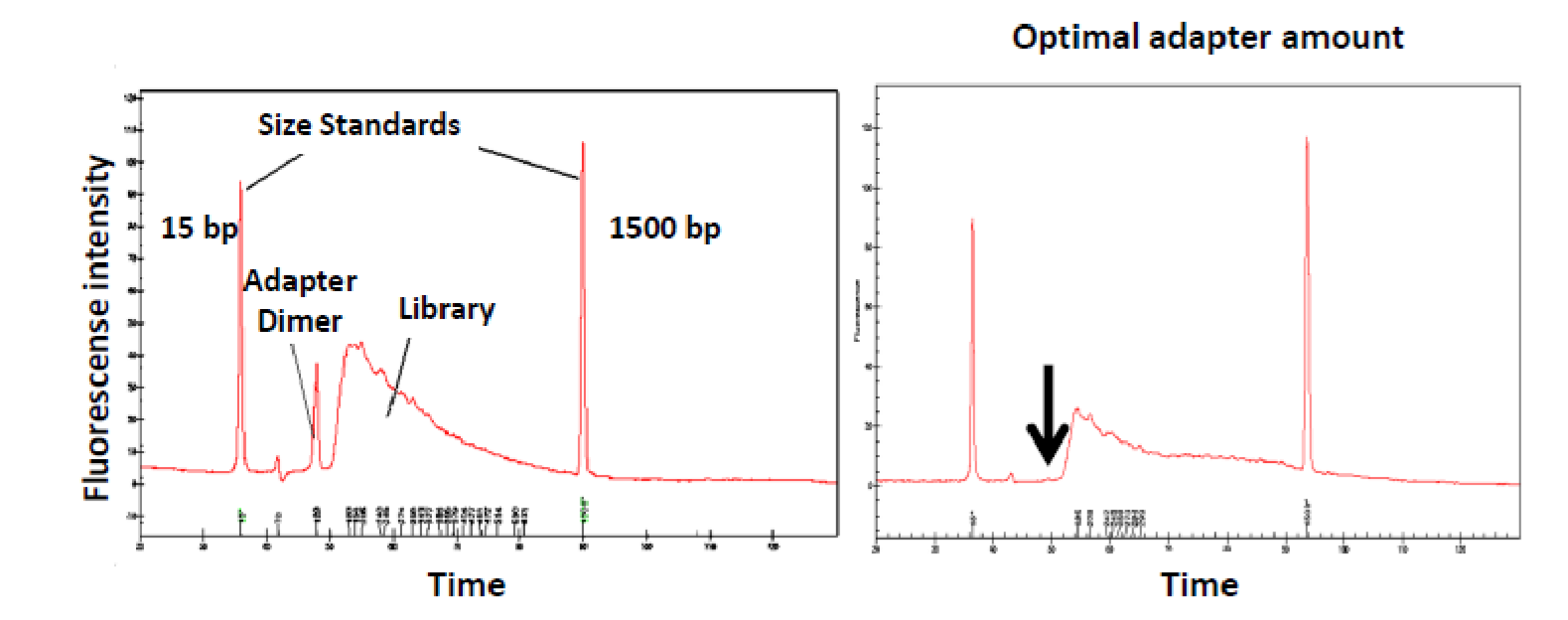


**Adapter Plate Setup**

24, 48, 96, 192 samples can be multiplexed together in a single lane of Illumina Hiseq sequencing library. One can do 96 samples at a time with an example adapter layout as under:


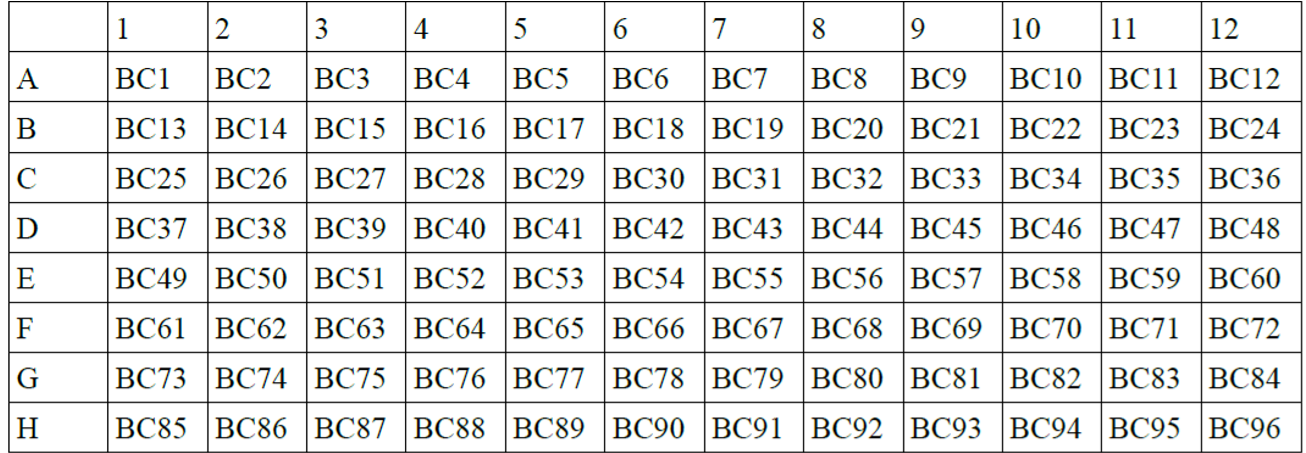

Supplement: Supplementary file 4 [file Data_Sheet_2.DOCX]
