# Supplementary material for: Mapping and QTL Analysis of Gynoecy and Earliness in Bitter Gourd (Momordica charantia L.) Using Genotyping-by-Sequencing (GBS) Technology
Source: Front Plant Sci. 2018 Oct 31;9:1555. doi: 10.3389/fpls.2018.01555 (PMC6220052; doi:10.3389/fpls.2018.01555)

**The analysis using UNEAK and work flow**

**Analysis using UNEAK (Universal Network Enabled Analysis Kit)**

The UNEAK is the non-reference Genotyping by Sequencing SNP calling pipeline, which is an extension of the Java program of TASSEL. UNEAK commands are run as TASSEL plugins via the command line in the following format (Linux or Mac operating systems).

**Workflow:**

**UNEAK Non** **-Reference based Flowchart:**

The flow chart below shows how the steps of the analysis link together. Blue boxes represent files produced at each step of the analysis, and Purple boxes represent the processes that produced them.


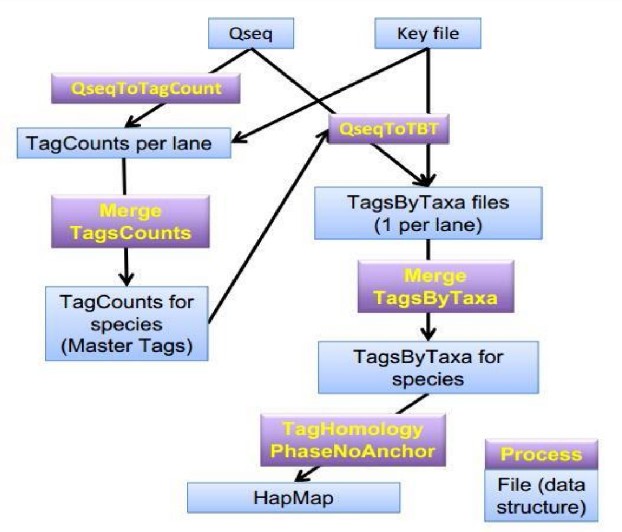


**A. FASTQ to TAG Count**

**1. Raw data QC and filtering**:

1. Start from unfiltered FASTQ reads. Because sometimes it discards good reads which map back perfectly to reference up to first 64 bases.
2. Starting with the FASTQ files from a flow cell, first filter for reads that

- perfectly matched one of the barcodes and the expected four-base remnant of the ENZYME cut site (For eg peK1 Site is CWGC),
- Were not adapter/adapter dimers, and
- Contained no ‘‘Ns’’ in their first 72 bases.

**2. Parsed file creation**:

- These reads are sorted into separate files (Using KEY FILE-Containing barcodes) according to their barcode, with the barcode removed and the remainder of the sequence trimmed to 64 bases (including the initial CUT SITE SEQUENCE CWGC).
- If either the full ENZYME site (from partial digestion or chimera formation) or the first 8 bases of common adapter (from ENZYME fragments less than 64 bases) were detected within 64 bases, the read was truncated appropriately and then filled to 64 bases with polyA.

**3**. To generate a set of UNIQUE 64 base sequence TAGS to be included in a presence/absence genotype table, only reads with a minimum Qscore of 10 across the first 72 bases) and that occurred at least twice are kept.

**4**. Counts how many times each unique 64 base read (tag) occurred in the FASTQ file.

**Pictorial Representation of a TYPICAL QC Filtering, Trimming & Unique Tags creation**


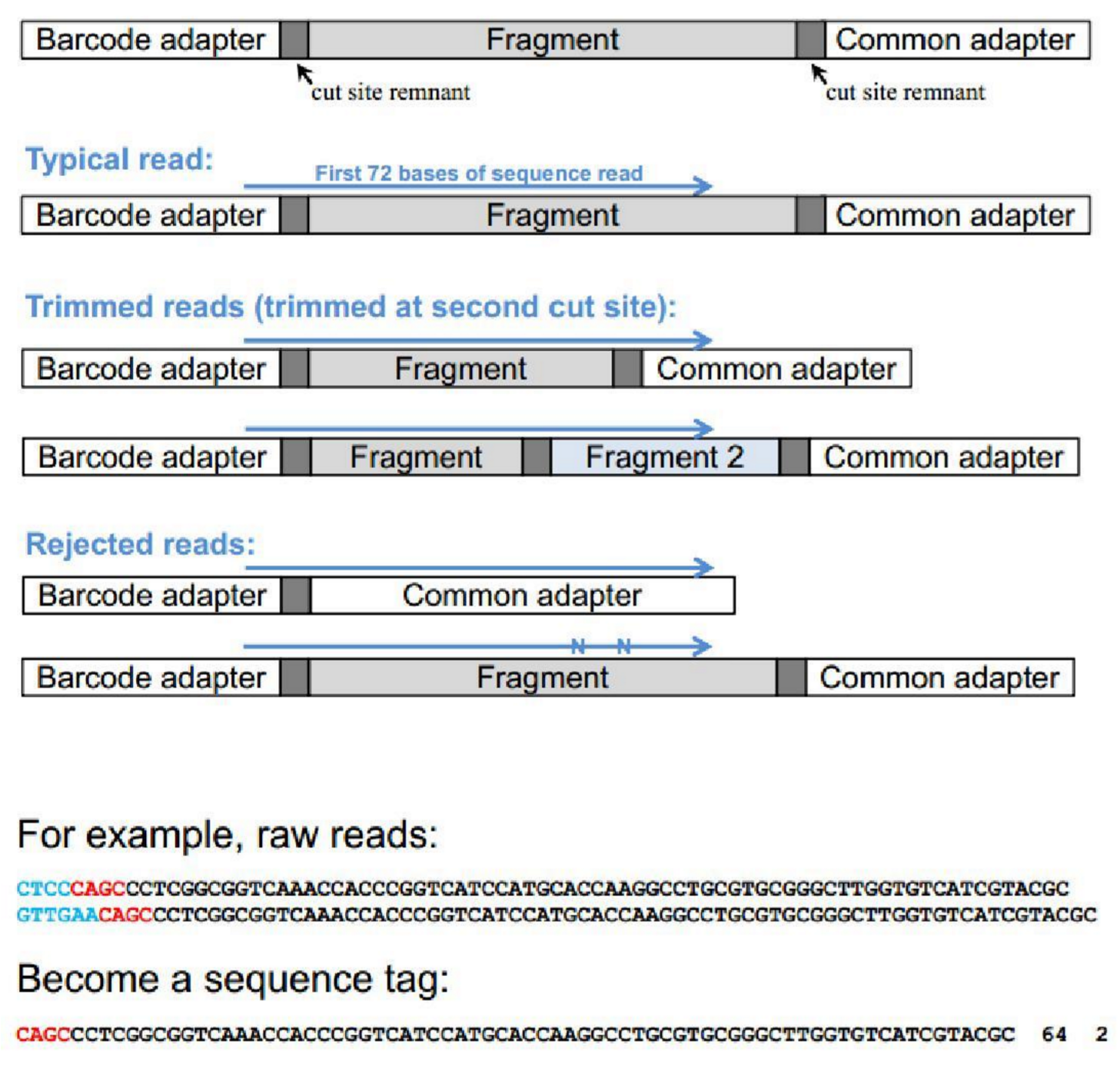


**An example of a TAG count file:**


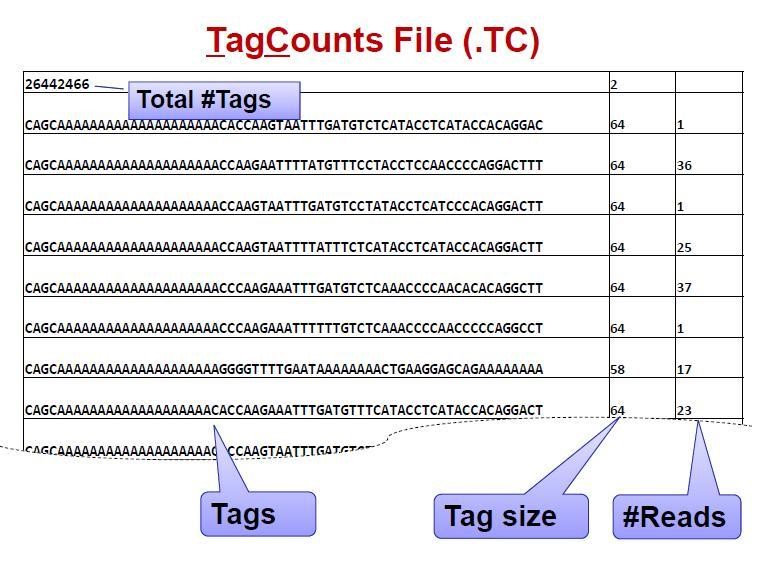


**B. FASTQ to TAGS by TAXA:**


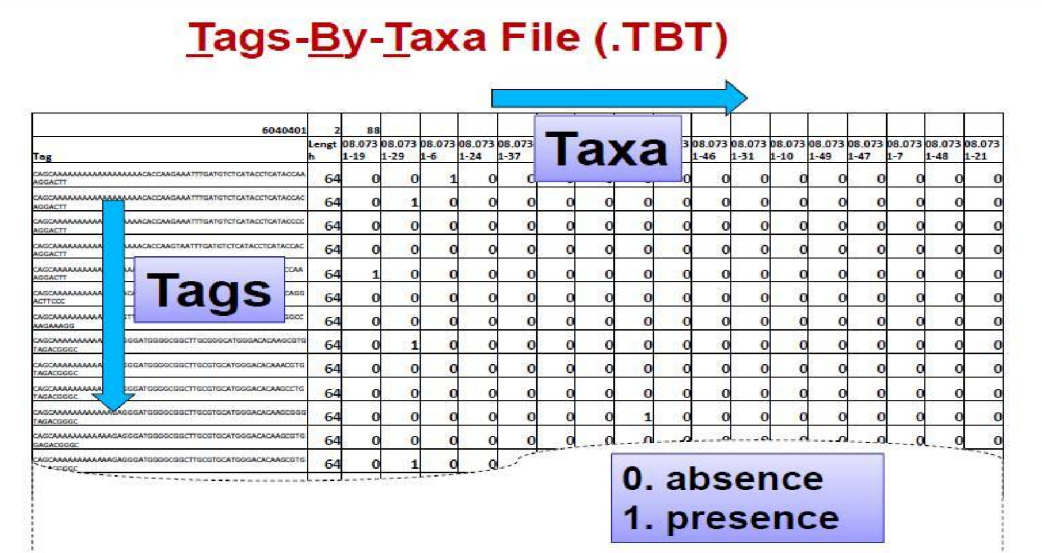


**Comparing different samples for presence or absence of tags with 0 as absence and 1 as presence of a tag.**

**C. Tag Data generation in HapMap format**

Using information from Tags by Taxa one can create a informative format of SNP report called HapMap format. The figure below is an example of the HapMap format:


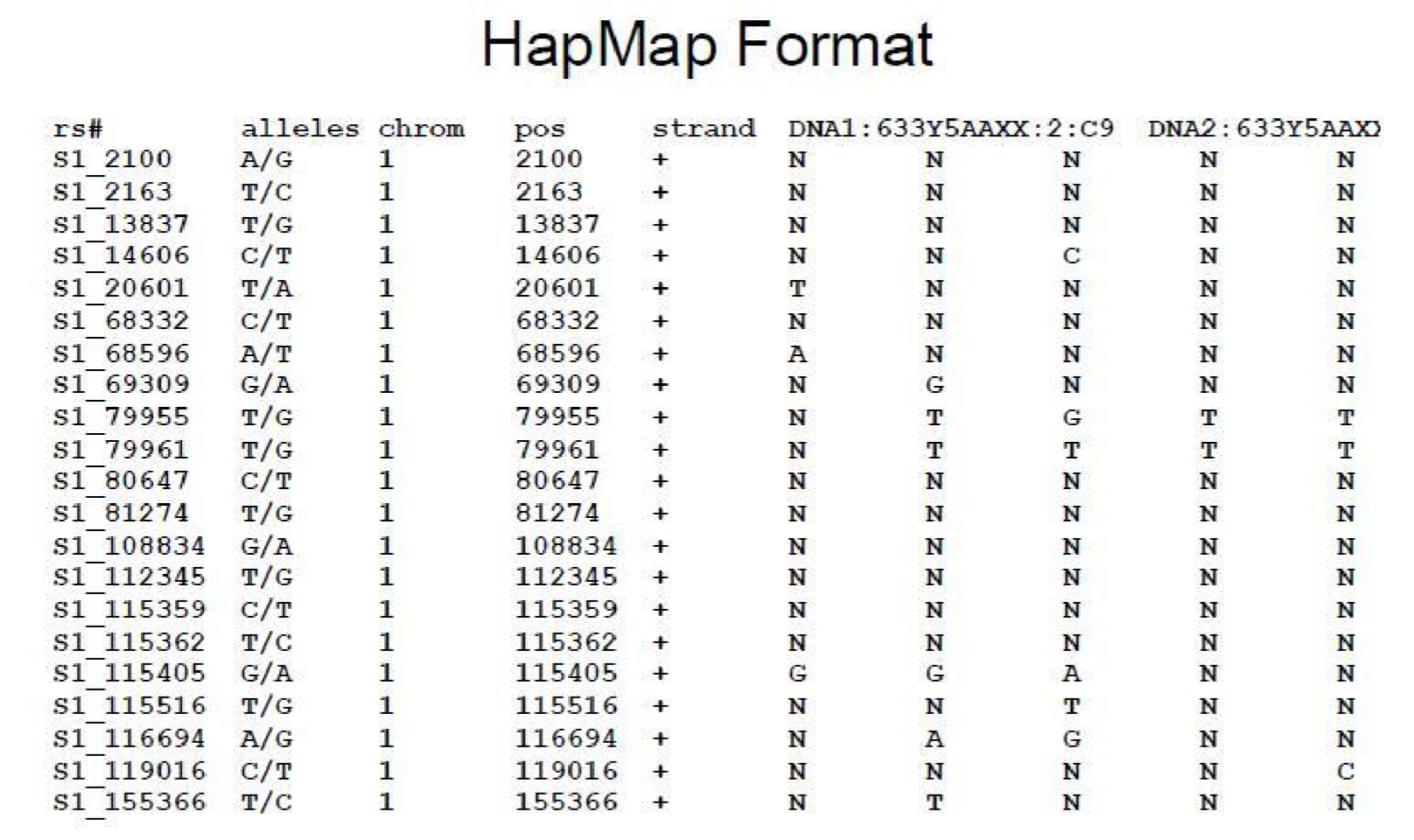

Supplement: Supplementary file 5 [file Data_Sheet_3.DOCX]
