# Supplementary material for: Mapping and QTL Analysis of Gynoecy and Earliness in Bitter Gourd (Momordica charantia L.) Using Genotyping-by-Sequencing (GBS) Technology
Source: Front Plant Sci. 2018 Oct 31;9:1555. doi: 10.3389/fpls.2018.01555 (PMC6220052; doi:10.3389/fpls.2018.01555)
Supplement: Supplementary file 6 [file Data_Sheet_4.DOCX]

**The summery of final library, sequencing report of UNEAK output file and allele summery**

**Sequencing Data Report:**

- Final library was sequenced using Illumina True Seq Version 4 **single end** Sequencing chemistry with read lengths of 150bp on Illumina Platform.
- The 94 Plex Library was sequenced on a **single lane** with read length of 150bp was used.

**RAW Data Statistics**:

| Properties | Bittergourd Fastq file |
| --- | --- |
| File Name (FASTQ format) | G2YYGACXX_1_fastq.txt |
| Total Data (in GB) | **18.4** |

**UNEAK OUTPUT:**

**Raw data Statistics:**

**Overall Summary**

| **Stat Type** | **Value** |
| --- | --- |
| Number of Taxa | 96 |
| Number of Sites | 93926 |
| Sites x Taxa | 9.02E+06 |
| Number Not Missing | 3.04E+06 |
| Proportion Not Missing | 0.3376 |
| Number Missing | 5.97E+06 |
| Proportion Missing | 0.6624 |
| Number Gametes | 1.80E+07 |
| Gametes Not Missing | 6.09E+06 |
| Proportion Gametes Not Missing | 0.3376 |
| Gametes Missing | 1.19E+07 |
| Proportion Gametes Missing | 0.6624 |
| Number Heterozygous | 271188 |
| Proportion Heterozygous | 0.03008 |

**Allele Summary**

| **Alleles** | **Number** | **Proportion** | **Frequency** |
| --- | --- | --- | --- |
| N | 5972798 | 0.6624 | 1.96209 |
| C | 780598 | 0.08657 | 0.25643 |
| G | 765845 | 0.08493 | 0.25158 |
| A | 619904 | 0.06875 | 0.20364 |
| T | 606563 | 0.06727 | 0.19926 |
| R | 90294 | 0.01001 | 0.02966 |
| Y | 88159 | 0.00978 | 0.02896 |
| W | 47063 | 0.00522 | 0.01546 |
| K | 18561 | 0.00206 | 0.0061 |
| M | 18372 | 0.00204 | 0.00604 |
| S | 8739 | 9.69E-04 | 0.00287 |
| C:T | 20578 | 0.21909 |  |
| G:A | 20034 | 0.2133 |  |
| A:G | 11903 | 0.12673 |  |
| T:C | 11497 | 0.1224 |  |
| A:T | 6254 | 0.06658 |  |
| T:A | 5743 | 0.06114 |  |
| G:T | 3818 | 0.04065 |  |
| C:A | 3626 | 0.0386 |  |
| A:C | 2852 | 0.03036 |  |
| T:G | 2698 | 0.02872 |  |
| C:G | 1793 | 0.01909 |  |
| G:C | 1554 | 0.01654 |  |
| G:G | 515 | 0.00548 |  |
| C:C | 468 | 0.00498 |  |
| T:T | 305 | 0.00325 |  |
| A:A | 288 | 0.00307 |  |

**Filtered File with 20% Minor Allele Frequency ( MAF), 40% missing data.**

**Over all Summary:**

| **Stat Type** | **Value** |
| --- | --- |
| Number of Taxa | 95 |
| Number of Sites | 4003 |
| Sites x Taxa | 380285 |
| Number Not Missing | 244848 |
| Proportion Not Missing | 0.64385 |
| Number Missing | 135437 |
| Proportion Missing | 0.35615 |
| Number Gametes | 760570 |
| Gametes Not Missing | 489696 |
| Proportion Gametes Not Missing | 0.64385 |
| Gametes Missing | 270874 |
| Proportion Gametes Missing | 0.35615 |
| Number Heterozygous | 0 |
| Proportion Heterozygous | 0 |

**Allele Summary**

| **Alleles** | **Number** | **Proportion** | **Frequency** |
| --- | --- | --- | --- |
| N | 135437 | 0.35615 | 0.55315 |
| A | 74455 | 0.19579 | 0.30409 |
| T | 73342 | 0.19286 | 0.29954 |
| G | 49411 | 0.12993 | 0.2018 |
| C | 47640 | 0.12527 | 0.19457 |
| C:T | 617 | 0.15413 |  |
| A:G | 592 | 0.14789 |  |
| G:A | 580 | 0.14489 |  |
| A:T | 561 | 0.14014 |  |
| T:C | 514 | 0.1284 |  |
| T:A | 508 | 0.1269 |  |
| G:T | 143 | 0.03572 |  |
| A:C | 125 | 0.03123 |  |
| T:G | 123 | 0.03073 |  |
| C:A | 113 | 0.02823 |  |
| C:G | 72 | 0.01799 |  |
| G:C | 55 | 0.01374 |  |

**Sample Summary:**

| **S/n** | **Individual** | **a** | **het** | **b** | **Missing data** | **Total Calls** |
| --- | --- | --- | --- | --- | --- | --- |
| 1 | 1 | 1053 | 0 | 831 | 1039 | 1884 |
| 2 | 2 | 1202 | 0 | 804 | 917 | 2006 |
| 3 | 3 | 1078 | 0 | 982 | 863 | 2060 |
| 4 | 4 | 939 | 0 | 1038 | 946 | 1977 |
| 5 | 5 | 942 | 0 | 932 | 1049 | 1874 |
| 6 | 6 | 1159 | 0 | 951 | 813 | 2110 |
| 7 | 7 | 1155 | 0 | 981 | 787 | 2136 |
| 8 | 8 | 1253 | 0 | 930 | 740 | 2183 |
| 9 | 9 | 1398 | 0 | 794 | 731 | 2192 |
| 10 | 10 | 1226 | 0 | 994 | 703 | 2220 |
| 11 | 11 | 1248 | 0 | 887 | 788 | 2135 |
| 12 | 12 | 1322 | 0 | 865 | 736 | 2187 |
| 13 | 13 | 1051 | 0 | 1038 | 834 | 2089 |
| 14 | 14 | 1255 | 0 | 890 | 778 | 2145 |
| 15 | 15 | 1235 | 0 | 982 | 706 | 2217 |
| 16 | 16 | 1048 | 0 | 1008 | 867 | 2056 |
| 17 | 17 | 1084 | 0 | 895 | 944 | 1979 |
| 18 | 18 | 1394 | 0 | 813 | 716 | 2207 |
| 19 | 19 | 1214 | 0 | 982 | 727 | 2196 |
| 20 | 20 | 1313 | 0 | 894 | 716 | 2207 |
| 21 | 21 | 1302 | 0 | 901 | 720 | 2203 |
| 22 | 22 | 1281 | 0 | 1007 | 635 | 2288 |
| 23 | 23 | 1081 | 0 | 1086 | 756 | 2167 |
| 24 | 24 | 1282 | 0 | 1008 | 633 | 2290 |
| 25 | 25 | 1254 | 0 | 934 | 735 | 2188 |
| 26 | 26 | 1235 | 0 | 1147 | 541 | 2382 |
| 27 | 27 | 1287 | 0 | 1072 | 564 | 2359 |
| 28 | 28 | 1284 | 0 | 1005 | 634 | 2289 |
| 29 | 29 | 1094 | 0 | 1116 | 713 | 2210 |
| 30 | 30 | 1462 | 0 | 932 | 529 | 2394 |
| 31 | 31 | 1333 | 0 | 947 | 643 | 2280 |
| 32 | 32 | 1379 | 0 | 868 | 676 | 2247 |
| 33 | 33 | 1323 | 0 | 979 | 621 | 2302 |
| 34 | 34 | 1153 | 0 | 954 | 816 | 2107 |
| 35 | 35 | 1362 | 0 | 1062 | 499 | 2424 |
| 36 | 36 | 1257 | 0 | 851 | 815 | 2108 |
| 37 | 37 | 1290 | 0 | 977 | 656 | 2267 |
| 38 | 38 | 1312 | 0 | 967 | 644 | 2279 |
| 39 | 39 | 1398 | 0 | 891 | 634 | 2289 |
| 40 | 40 | 1180 | 0 | 1053 | 690 | 2233 |
| 41 | 41 | 1091 | 0 | 1060 | 772 | 2151 |
| 42 | 42 | 1189 | 0 | 854 | 880 | 2043 |
| 43 | 43 | 1297 | 0 | 906 | 720 | 2203 |
| 44 | 44 | 1188 | 0 | 817 | 918 | 2005 |
| 45 | 45 | 1281 | 0 | 1047 | 595 | 2328 |
| 46 | 46 | 1110 | 0 | 888 | 925 | 1998 |
| 47 | 47 | 1384 | 0 | 946 | 593 | 2330 |
| 48 | 48 | 1273 | 0 | 958 | 692 | 2231 |
| 49 | 49 | 1011 | 0 | 1006 | 906 | 2017 |
| 50 | 50 | 1255 | 0 | 942 | 726 | 2197 |
| 51 | 51 | 1406 | 0 | 848 | 669 | 2254 |
| 52 | 52 | 1253 | 0 | 952 | 718 | 2205 |
| 53 | 53 | 1170 | 0 | 887 | 866 | 2057 |
| 54 | 54 | 1030 | 0 | 732 | 1161 | 1762 |
| 55 | 55 | 1295 | 0 | 961 | 667 | 2256 |
| 56 | 56 | 1408 | 0 | 924 | 591 | 2332 |
| 57 | 57 | 1113 | 0 | 819 | 991 | 1932 |
| 58 | 58 | 1349 | 0 | 959 | 615 | 2308 |
| 59 | 59 | 1254 | 0 | 861 | 808 | 2115 |
| 60 | 60 | 1286 | 0 | 1051 | 586 | 2337 |
| 61 | 61 | 1209 | 0 | 721 | 993 | 1930 |
| 62 | 62 | 1287 | 0 | 896 | 740 | 2183 |
| 63 | 63 | 1312 | 0 | 991 | 620 | 2303 |
| 64 | 64 | 1076 | 0 | 862 | 985 | 1938 |
| 65 | 65 | 1253 | 0 | 983 | 687 | 2236 |
| 66 | 66 | 1313 | 0 | 858 | 752 | 2171 |
| 67 | 67 | 1122 | 0 | 1078 | 723 | 2200 |
| 68 | 68 | 1409 | 0 | 872 | 642 | 2281 |
| 69 | 69 | 1326 | 0 | 857 | 740 | 2183 |
| 70 | 70 | 864 | 0 | 723 | 1336 | 1587 |
| 71 | 71 | 1288 | 0 | 885 | 750 | 2173 |
| 72 | 72 | 1473 | 0 | 822 | 628 | 2295 |
| 73 | 73 | 1231 | 0 | 1075 | 617 | 2306 |
| 74 | 74 | 1313 | 0 | 928 | 682 | 2241 |
| 75 | 75 | 1280 | 0 | 768 | 875 | 2048 |
| 76 | 76 | 1290 | 0 | 862 | 771 | 2152 |
| 77 | 77 | 1254 | 0 | 1026 | 643 | 2280 |
| 78 | 78 | 1320 | 0 | 924 | 679 | 2244 |
| 79 | 79 | 1167 | 0 | 987 | 769 | 2154 |
| 80 | 80 | 208 | 0 | 86 | 2629 | 294 |
| 81 | 81 | 204 | 0 | 115 | 2604 | 319 |
| 82 | 82 | 204 | 0 | 99 | 2620 | 303 |
| 83 | 83 | 225 | 0 | 81 | 2617 | 306 |
| 84 | 84 | 205 | 0 | 61 | 2657 | 266 |
| 85 | 85 | 190 | 0 | 93 | 2640 | 283 |
| 86 | 86 | 217 | 0 | 90 | 2616 | 307 |
| 87 | 87 | 1152 | 0 | 1110 | 661 | 2262 |
| 88 | 88 | 1203 | 0 | 905 | 815 | 2108 |
| 89 | 89 | 1182 | 0 | 1019 | 722 | 2201 |
| 90 | 90 | 1300 | 0 | 942 | 681 | 2242 |
| 91 | DBGy-201 | 1274 | 0 | 888 | 761 | 2162 |
| 92 | PDM | 1199 | 0 | 1089 | 635 | 2288 |
| 93 | F_1_ | 1261 | 0 | 973 | 689 | 2234 |
